# Imparting Water Solubility and Aqueous Electrochemical Activity to Ferrocene upon Confinement

**DOI:** 10.1021/acs.inorgchem.6c01327

**Published:** 2026-04-29

**Authors:** Ryan J. Bujol, Nathan H. Mitchell, Siddhiaratchige D. M. Siddhiaratchi, Thomas K. Weldeghiorghis, Frank R. Fronczek, Noémie Elgrishi

**Affiliations:** Department of Chemistry, 5779 Louisiana State University, 232 Choppin Hall, Baton Rouge, Louisiana 70803, United States

## Abstract

Electrocatalytic processes underpin technological advances in areas ranging from energy conversion and storage to electrosynthesis of fine and commodity chemicals. Catalyst confinement is an attractive avenue to gain control over activity and selectivity, as well as to tailor solubility or decrease deactivation. The use of supramolecular materials as hosts to confine and stabilize electroactive complexes would be an exciting prospect. Here we report the impact of confinement on the solubility and electrochemical properties of ubiquitous electrochemical probe ferrocene. The notorious water insolubility of ferrocene is overcome through confinement within a Ga_4_L_6_ supramolecular cage, and the new host–guest complex not only retains the electroactivity of the incorporated ferrocene molecule but also decreases the oxidation potential of ferrocene by 200 mV, rendering it easier to oxidize. In the context of an electrocatalytic oxidation, this would translate to unlocking activity in a green solvent while reducing the overpotential for catalysis. This work serves as a proof-of-concept demonstration of the potential of supramolecular coordination cages as confinement vessels for molecular electrocatalysts.

## Graphical abstract

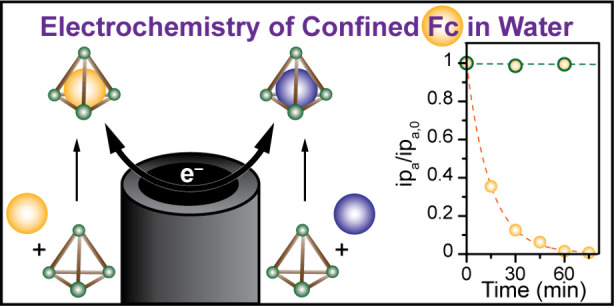

## Introduction

Molecular electrocatalysts have emerged as important tools for green chemistry as they can use electricity, generated by solar, renewables, and other carbon-efficient sources, to produce fuels or other chemicals in sustainable ways.^1–3^ Although many examples of molecular electrocatalysts exist that are highly active under cyclic voltammetry conditions, bulk-scale applications can reveal degradation pathways that are unknown on the laboratory scale.^4^ Methods to stabilize these systems are well-studied, and can involve modifying the ligand environment of the catalyst to reduce deleterious activity, immobilizing the catalyst on a substrate surface to discourage physical interactions between active centers, or confinement inside porous supramolecular materials.^5–14^ The latter represents a particularly exciting and unique approach, as no structural modifications to the catalyst are required, which might otherwise affect catalytic efficiency. However, for electrocatalysts specifically, the need for electron transfer to confined catalysts makes this method challenging as the nature of the confinement could simultaneously present an insulating environment. Supramolecular metal–organic cages could be a promising avenue to tackle this challenge, while at the same time offering a simple path to tailor the solubility of electrocatalysts. For this to be accomplished, data are needed to understand the impact of catalyst confinement on electrochemical properties. There is also a dearth of data on the electrochemical properties of supramolecular metal–organic systems.^15–17^ To establish whether porous supramolecular systems are suitable for preventing the deactivation of encapsulated electrocatalysts, a foundational study that assesses the impact of confinement on the electrochemical properties of a known stable redox probe is first required. This work describes an electron transfer from an electrode to a metal complex confined within a metal–organic cage where confinement is maintained after the electron transfer. To our knowledge, this is the first such example. We report the preparation and electrochemical properties of an inclusion complex of a molecule of ferrocene (Cp_2_Fe, Fc) encapsulated in the cavity of a K_12_[Ga_4_L_6_][Bibr ref18] supramolecular coordination cage (Scheme 1). The impact of Fc confinement on its solubility in water, retention in water, and electrochemical properties is studied.

**Scheme 1:**
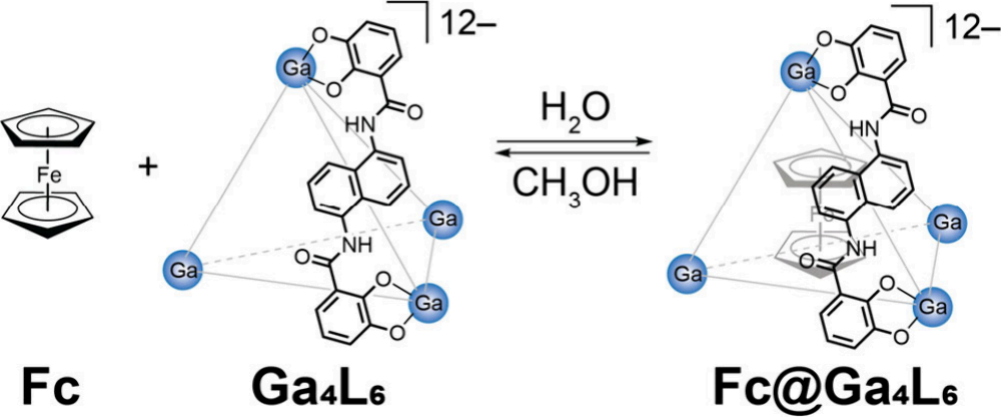
Formation of Host–Guest Complex Fc@Ga_4_L_6_ in Water^a^

a In methanol, the Fc molecule departs from the cavity.

The K_12_[Ga_4_L_6_] supramolecular coordination cage chosen for this study was first reported by the group of Raymond and collaborators.^18^ This nanocontainer has been extensively studied for the encapsulation of a variety of cationic guests.^17,19,20^ Excellent host–guest properties are reported, in particular toward monocationic guests. The molecular cage with K^+^ counterions is also soluble and stable in water at near-neutral pH, and possesses a hydrophobic inner cavity. Fc was selected as the electrochemical probe in this study due to its ubiquitous use in electrochemical systems in general, despite its known insolubility in water.

## Results and Discussion

### Formation of the Host–Guest Complex

When 1 equiv of Fc was added to a solution of K_12_[Ga_4_L_6_] in D_2_O, no reaction took place initially. This is expected as the solubility of Fc in water is extremely low.^21,22^ Upon mixing, the solution became clear, with the uptake of Fc suggesting the formation of the inclusion complex Fc@Ga_4_L_6_ (Scheme 1). This is supported by ^1^H NMR spectroscopic data (Figure 1). Indeed, a shift of the ^1^H NMR resonances of the host is observed, which suggests interaction with another species in solution. This is accompanied by the appearance of a singlet at 0.76 ppm integrating for 10 H per molecular cage, as would be expected for the encapsulation of one Fc molecule per cage. The singlet attributed to Fc is significantly shifted compared to other known water-soluble cyclopentadienyl resonances. Resonances in D_2_O associated with water-soluble ferrocene derivatives functionalized with alkylammonium groups are reported at 3.76 and 4.66 ppm.^23–26^ Estimates of chemical shifts for the ^1^H NMR resonance of Fc in D_2_O, in a saturated Fc solution, resulted in a small singlet at 4.3 ppm (Figure S1), further supporting that the observed shift to 0.76 ppm and increased intensity in the presence of the Ga_4_L_6_ metal–organic cage are indicative of encapsulation of Fc.

**Figure 1:**
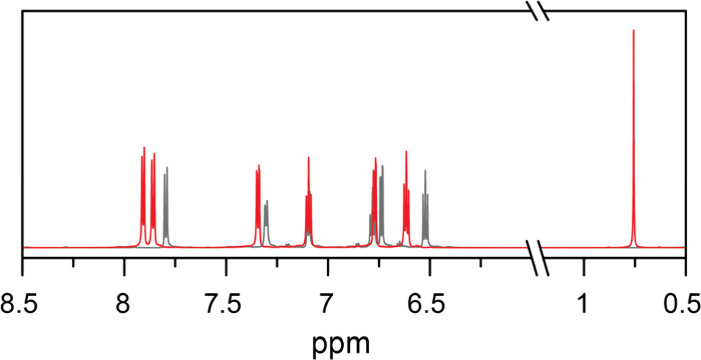
^1^H NMR spectra of the Fc@Ga_4_L_6_ complex (red) and the empty Ga_4_L_6_ cage (gray) in D_2_O (700 MHz). The Fc resonance at 0.76 ppm integrates for 10 protons per cage.

The independent synthesis of Fc@Ga_4_L_6_ was carried out by adding excess Fc to an aqueous solution of K_12_[Ga_4_L_6_] in a sealed flask under N_2_, and placing the flask in an ultrasonic bath for 60 min. The reaction was then filtered under N_2_ to remove the excess Fc. The solvent was removed under vacuum, yielding a golden-brown solid which was characterized by ^1^H, ^13^C, HSQC and NOESY NMR spectroscopy techniques (data and assignments provided in Figures S2–S5). Single crystals of the resulting product were grown under N_2_ by slow evaporation of an aqueous solution (Figure S6). The orange crystals were suitable for X-ray crystallography at a resolution of 0.84 Å, which was sufficient to establish connectivity. The structure, containing numerous disordered solvent molecules, confirmed the encapsulation of one Fc molecule within the cavity of the cage (Figure 2), though the data quality is not sufficient to determine precise bond distances. While the X-ray structure confirms the uptake of Fc into the cavity of the cage in the solid state, it also further strengthens the assignment of Fc confinement within the cage in water. Though the Ga_4_L_6_ structure is typically known to encapsulate monocationic species through electrostatic interactions thanks to the overall −12 charge, the hydrophobicity of the cavity is a likely driver of the solubilization and confinement of the Fc molecule. To further probe the interactions of Fc with the Ga_4_L_6_ structure, Diffusion-Ordered Spectroscopy (DOSY) data were collected in both D_2_O and in CD_3_OD to obtain estimates of diffusion coefficients for Fc and for Ga_4_L_6_ (Figures S7–S13). The data summarized in Table 1 show that in samples of Ga_4_L_6_ in water, with or without KCl present, the diffusion coefficients obtained for the Ga_4_L_6_ resonances match the value obtained for the confined Fc signal. This indicates that the two species diffuse at the same rate, further supporting confinement of Fc within the Ga_4_L_6_ structure. In contrast, when a sample of Fc@Ga_4_L_6_ is dissolved in CD_3_OD, the diffusion coefficients obtained for Fc and for Ga_4_L_6_ differ by close to an order of magnitude, matching the values obtained for independent samples of Fc in CD_3_OD and for Ga_4_L_6_ in CD_3_OD, respectively. The difference in diffusion coefficients indicates that the strong interaction observed in D_2_O does not take place in methanol, a solvent in which Fc is soluble. This is further supported by the lack of shifts of NMR resonances of Fc or Ga_4_L_6_ when both are present in CD_3_OD (Figures S11–13).

**Figure 2:**
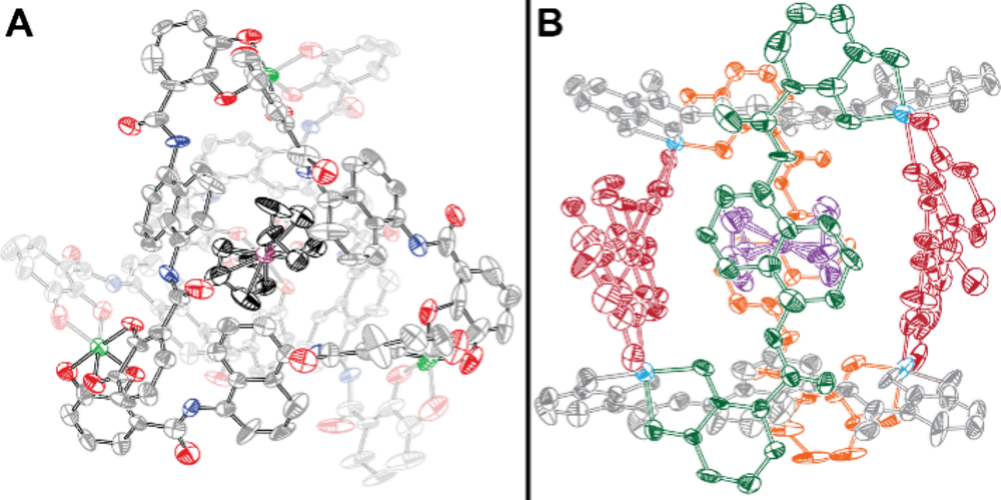
Crystal structure of Fc@Ga_4_L_6_. Ellipsoids drawn at 50% probability. Counter cations (12 K^+^) and hydrogen atoms have been omitted for the sake of clarity. (A) Atom color assignments: gray or black for C, blue for N, red for O, green for Ga, and purple for Fe. (B) Different view with Fc colored purple, Ga colored light blue, and ligands color-coded for the sake of clarity (front in green, back in orange, sides in red, and top and bottom in gray).

**Table 1:** Summary of *D*_0_ Values Obtained through DOSY

	*D*_0_ (×10^–6^ cm^2^ s^–1^)		
solvent	Fc@Ga_4_L_6_	K_12_[Ga_4_L_6_]	Fc
D_2_O	2.4 for Ga_4_L_6_; 2.4 for Fc	2.4	^a^
D_2_O, 1 M KCl	3.0 for Ga_4_L_6_; 3.0 for Fc	3.6	^a^
CD_3_OD	5.8 for Ga_4_L_6_; 29 for Fc	5.6	26

a The lack of solubility prevents data collection.

Overall, these data support that the confinement of Fc within the cavity of the Ga_4_L_6_ structure observed in the solid state is maintained in solution. It is noteworthy that the encapsulation of a neutral species such as Fc can occur within Ga_4_L_6_ as this host is known for encapsulating cationic species in general, with a preference for monocationic species.^27^ To the best of our knowledge, prior examples of neutral species bound within Ga_4_L_6_ all involve organic compounds, such as cycloalkanes, benzamides, and arenes.^28–30^ One example of weakly binding zwitterionic species has been reported.^20^ To date, no other neutral organometallic species are known to strongly encapsulate within Ga_4_L_6_.

### Impact of Confinement on Electrochemical Properties

As established, there is a strong driver for the confinement of the neutral molecule Fc within the Ga_4_L_6_ structure in water. Once confined, no evidence of release has been observed. This, coupled to the known even stronger affinity of the Ga_4_L_6_ structure toward encapsulating monocationic guests, makes this Fc@Ga_4_L_6_ host–guest complex poised to provide an ideal system to study the electrochemical properties of a redox active probe under confinement (Scheme 2, bottom).

**Scheme 2:**
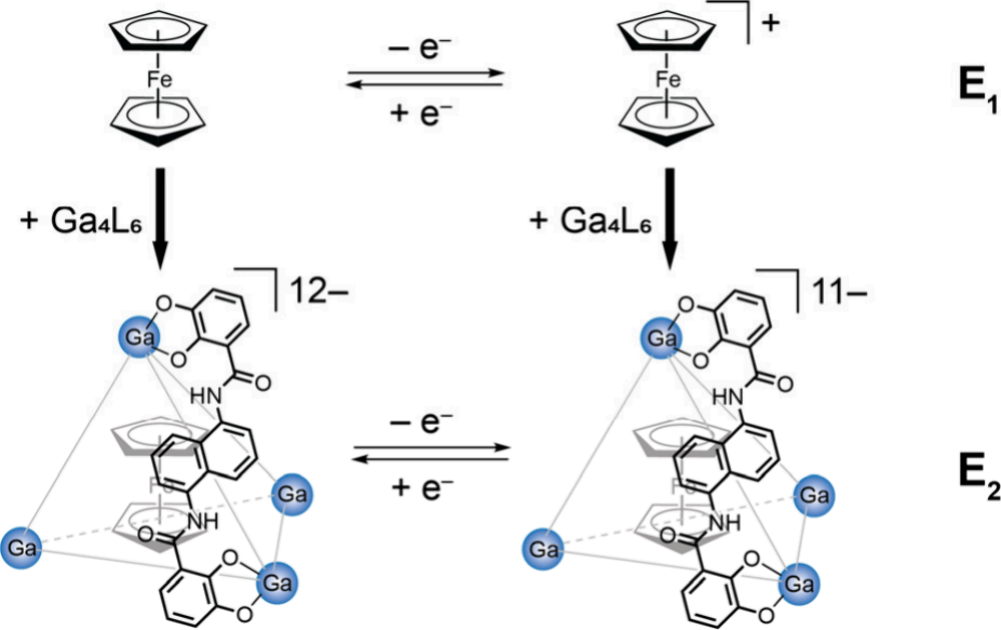
Electrochemical Activity of Free Fc in Solution (E_1_, top) and of Fc@Ga_4_L_6_ (E_2_, bottom)

Indeed, the oxidation of Fc to Fc^+^ in water provides a unique opportunity to study an electrochemical system in which the release of the confined guest is even more disfavored upon electron transfer.

The electrochemical properties of Fc upon encapsulation within K_12_[Ga_4_L_6_] were thus investigated using cyclic voltammetry. Cyclic voltammograms (CVs) of the host K_12_[Ga_4_L_6_] alone show that the empty cage is stable and inert in an electrochemical cell across a wide range of potentials in water (Figure S14). The CV of K_12_[Ga_4_L_6_] was characterized by an extended potential window, from 0.2 V to the onset of water reduction near −1.5 V vs Ag/AgCl, in which no Faradaic responses are observed (Figure S14). When scanned sufficiently positive, irreversible oxidative breakdown of the cage occurs, with two large waves at 0.26 and 0.53 V vs Ag/AgCl being observed and two smaller reductive features on the return trace (Figure S14). In contrast, CVs of the Fc@Ga_4_L_6_ complex (Figure 3, red trace) show a quasi-reversible wave centered at *E*_1/2_ = −0.037 V vs Ag/AgCl with a peak-to-peak separation of 91 mV at 100 mV s^–1^.

**Figure 3:**
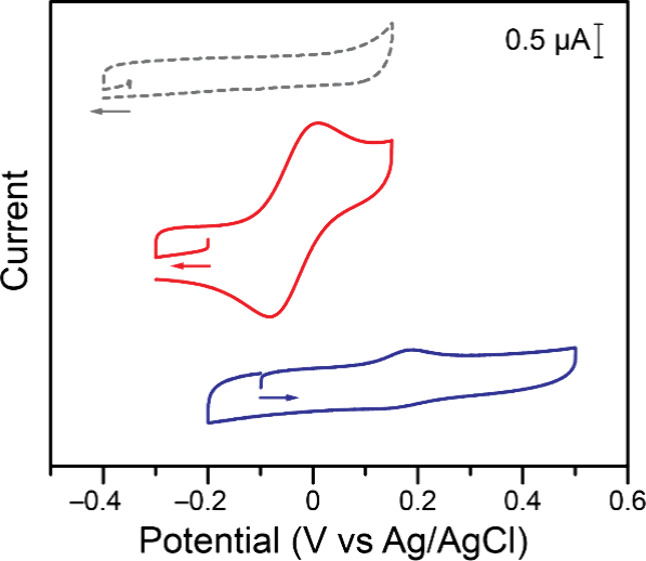
Cyclic voltammograms of 0.250 mM Ga_4_L_6_ (gray), 0.250 mM Fc@Ga_4_L_6_ (red), and a saturated solution of Fc (blue). Data recorded at 100 mV s^–1^ on a 3 mm diameter glassy carbon working electrode, in water with 1 M KCl.

Given the lack of electrochemical features for Ga_4_L_6_ in this potential range (gray trace in Figure 3), this electrochemical feature is assigned to the confined Fc molecule, corresponding to E_2_ in Scheme 2. The feature does not change upon the addition of excess K_12_[Ga_4_L_6_], and it is notably shifted much more negative, *i.e*. Fc is easier to oxidize, compared to the ill-defined signals observed for Fc in water (blue trace in Figure 3).

The observation of the new electrochemical feature for the confined ferrocene is promising, as several reports note the inhibition of the electrochemical activity of Fc upon interaction with some supramolecular structures. The group of Angel Kaifer in particular reported that the interaction of Fc with an organic cavitand from the Rebek group prevented electrochemical activity in CVs collected in water.^31^ This was also true of Cp_2_Co^+^ and Fc­(CH_2_N­(CH_3_)_3_)^+^.^31^ Within Gibb’s octa-acid cavitand, Fc again exhibits no voltammetric response in aqueous solution.^32^ However, Fc­(CH_2_N­(CH_3_)_3_)^+^ added as an external redox-mediator was shown to shuttle electrons to the encapsulated Fc.^32^ Thus, the electrochemical behavior of Fc could be indirectly observed in this system. In comparison, our work circumvents these issues, allowing for direct observation of the redox behavior of Fc in aqueous solutions without the need for a mediator.

Beyond the observation of the retention of electrochemical activity for Fc in water under confinement, the data in Figure 3 point to a 200 mV negative shift between the electrochemical feature observed in the absence and presence of the Ga_4_L_6_ structure (E_1_ vs E_2_ in Scheme 2). Other shifts have been reported for host–guest systems, depending on the properties of the host, the guest, and the solvent. For example, in the ring-shaped neutral host curcurbit[7]­uril (CB7), both cationic and neutral derivatives of ferrocene showed shifted *E*_1/2_ values for the Fc cores, with the cationic guests experiencing a larger shift of up to +110 mV.^33^ Similar observations have been reported in organic solvents with other organic hosts.^34–36^ Contrary to the data obtained for Fc@Ga_4_L_6_, the reported shifts are positive, meaning the oxidations of the metallocenes are hindered by the presence of the CB7 host. In further contrast with the Fc@Ga_4_L_6_ system herein, the authors also note that there is a distinct decrease in the stabilization interactions between CB7 and the functionalized Fc derivatives studied upon oxidation.^33^ When focusing on metal–organic cages as hosts, there is a dearth of reports to compare to. However, a report from the Fujita group parallels the present work with the use of a Pd_4_L_6_ cage encapsulating 4 Fc molecules in the solid state.^37^ The Pd_4_L_6_ structure is cationic, charged +12, with large pore openings on alternating faces of an octahedron. In CVs in water, the electrochemical signal for Fc is shifted positively by 73 mV. The authors attribute this shift to the cationic nature of the cage, which disfavors the binding of Fc^+^. This behavior is opposite to the Fc@Ga_4_L_6_ system, which has a −12 charge and leads to a 200 mV negative shift of E_2_ in Scheme 2 for Fc under confinement. This further supports that the observed behavior may be attributed to the highly anionic nature of the cage. The charge of the host, and thus the stabilization of the Fc moiety before and after the electron transfer, dictates the direction and magnitude of the shift in *E*_1/2_ observed. In the context of electrocatalysis, a diminution of the required overpotential could be achieved through careful matching of the properties of the catalyst and host.

To further characterize the electrochemical properties, a variable scan rate study was also conducted on the Fc@Ga_4_L_6_ complex, and the experiment was repeated three times (representative data set and analysis in Figures S15–S18). The Faradaic contributions to the anodic and cathodic peak currents were measured at all scan rates, normalized to the analyte concentration, and plotted as a function of the square root of the scan rate (Figure 4, left). Linear fits to both the anodic and cathodic peak currents confirmed that the analyte follows the Randles–Ševčík equation, indicating a diffusion-controlled electron transfer with no indication of deposition on the surface of the electrode.

**Figure 4:**
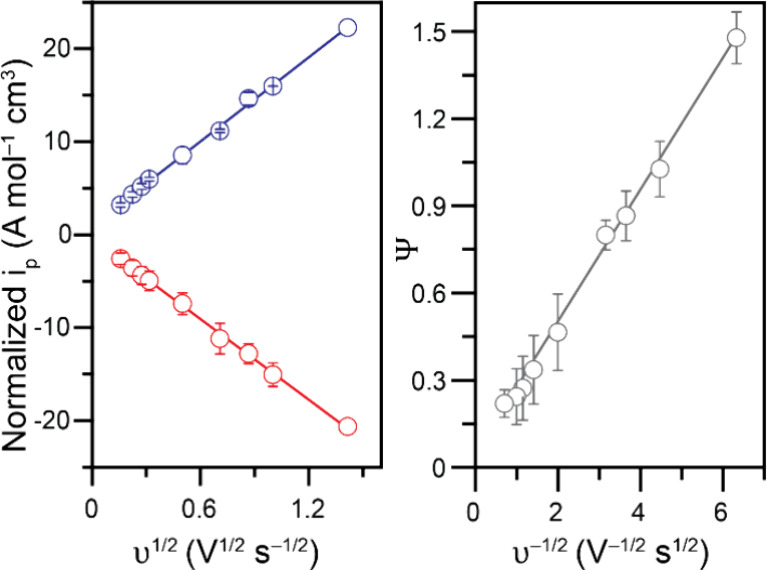
Randles–Ševčík (left) and Nicholson (right) analysis of a variable scan rate study of 0.25 mM Fc@Ga_4_L_6_ in 1 M KCl in water. Data recorded at various scan rates on freshly polished 3 mm diameter glassy carbon working electrodes.

Using the Randles–Ševčík equation, diffusion coefficients were calculated for the oxidation and reduction features observed for Fc@Ga_4_L_6_. The linear fits for the normalized Faradaic peak currents in Figure 4 (left) resulted in slopes of 15.1 A mol^–1^ cm^3^ V^–1/2^ s^1/2^ (r^2^ = 0.998) for the oxidation and of −14.5 A mol^–1^ cm^3^ V^–1/2^ s^1/2^ (r^2^ = 0.998) for the reduction. This yielded an average diffusion coefficient for the anodic data of *D*_*0a*_ = 6.32 × 10^–7^ cm^2^ s^–1^ while the cathodic peak gave an average value of *D*_*0c*_ = 6.36 × 10^–7^ cm^2^ s^–1^. The overall average was calculated at *D*_0_ = (6.34 ± 0.12) × 10^–7^ cm^2^ s^–1^ in these conditions, which lies within an order of magnitude of the values obtained through DOSY experiments.

In an attempt to compare the electrochemical properties determined for the confined Fc within Ga_4_L_6_ to those of free Fc in water, cyclic voltammetry data were collected for a filtered saturated solution of Fc in 1 M KCl (Figures S20–S22). Similarly to the blue trace in Figure 3, the CVs showed a redox couple with an apparent *E*_1/2_ of 0.163 V vs Ag/AgCl at 100 mV s^–1^ with a peak-to-peak separation of only 43 mV and an overall shape indicating surface adsorption. This is in good agreement with prior reports in related conditions.^37^ To moderate the effect of deposition and local accumulation on the electrode surface of the nearly insoluble Fc in 1 M KCl, the electrode was rinsed with CH_3_CN followed by water, and dried under N_2_ between each scan. CVs collected while varying the scan rate confirm that the electron transfer does not behave as a reversible Nernstian diffusion-controlled electron transfer typically observed for Fc in organic solvents. Instead, a shrinking of the peak-to-peak separation occurs as the scan rate is increased (Figure S21), in direct contrast to what is observed for the Fc@Ga_4_L_6_ data. To further compare with the Fc@Ga_4_L_6_ data, the evolution of the peak currents was plotted as a function of the square-root of the scan rate (Figure S22). The anodic peak currents do not track linearly with the square root of the scan rate, further indicating that the oxidation of Fc in water does not follow the Randles–Ševčík equation. Diffusion coefficient values of 5.1 × 10^–6^ cm^2^ s^–1^ and 6.7 × 10^–6^ cm^2^ s^–1^ have been reported using electrochemical methods in water for FcCH_2_COO^–^ and FcCOO^–^ respectively, which are water-soluble carboxylate derivatives of ferrocene.^38,39^ Critically, the diffusion coefficient values of these derivatives are an order of magnitude higher than the value calculated for Fc@Ga_4_L_6_ using electrochemical methods. A decrease in the diffusion coefficient upon confinement, which is also in agreement with the DOSY data, can be explained by the larger hydrodynamic radius of Fc@Ga_4_L_6_ compared to free Fc. Similar observations have been reported for other host–guest systems.^32,36,40–45^

The scan rate dependence data discussed above (Figure 4) was also used to calculate the heterogeneous electron transfer rate constant *k*_0_ using the Nicholson method (representative data set and analysis in Figure S17). The analysis of the experiment, performed in triplicate (Figure 4, right), resulted in a linear fit with a slope of 0.227 V^1/2^ s^–1/2^ (r^2^ = 0.995). This yielded an average heterogeneous electron transfer rate constant of *k*_0_ = (2.04 ± 0.06) × 10^–3^ cm s^–1^ for this electrochemical feature. The evolution of peak-to-peak separation with scan rate was also analyzed using the “trumpet plot” method (Figure S19) using a working curve of a simulation of a one-electron transfer redox couple with known *D*_0_ and *k*_0_ values to estimate the value of the heterogeneous electron transfer rate constant.^46^ Using this method, an average value of *k*_0_ = 2.24 × 10^–3^ cm s^–1^ was obtained, which is in good agreement with the value obtained from the Nicholson method. As the electron transfer for free Fc was not diffusion-controlled (Figures S20–S22), the Nicholson method could not be used to calculate a *k*_0_ for free Fc in water. As points of comparison, a prior report estimates a *k*_0_ value of 0.01 cm s^–1^ for free Fc in NEt_4_ClO_4_ on a platinum working electrode,^47^ which is in line with values in organic media on glassy carbon electrodes.^48^ These values would suggest that confinement of Fc within the Ga_4_L_6_ cage slows down the electron transfer. Similar decreases in *k*_0_ have been reported for Fc encapsulated in a β-cyclodextrin polymer^49^ and for ferrocene derivatives bound within systems such as organic hemicarcerands or CB7.^33,34^ A report from the Reek group notes that functionalization of Pd-based nanospheres with guanidinium-sulfonate Fc units attenuates the heterogeneous electron transfer rate constants of the appended Fc units by 20 to 250-fold.^50^ These results further support the observe attenuation of *k*_0_ for Fc upon confinement within the Ga_4_L_6_ structure.

To further confirm that the redox couple observed for Fc@Ga_4_L_6_ was due to Fc encapsulated in Ga_4_L_6_, CVs were collected of *in situ* generated Fc@Ga_4_L_6_. First, CVs were collected of excess free Fc in 1 M KCl to establish the peak currents and positions of free Fc. Enough K_12_[Ga_4_L_6_] was then added to the solution to give a Ga_4_L_6_ concentration of 0.25 mM. The solution was then stirred, and CVs were collected again. The data confirm that it matches presynthesized samples (Figure S23).

Overall, the data demonstrate the modulation of the electrochemical properties of Fc upon confinement within Ga_4_L_6_. The Fc/Fc^+^ redox couple becomes pseudoreversible and Nernstian in water under confinement. The oxidation becomes thermodynamically easier as evidenced by the shift in *E*_1/2_, but both the diffusion coefficient and the heterogeneous electron transfer rate constant decrease upon confinement.

### Solvation of Fc in Water

The data discussed above (Figure S23) also emphasize the increase in the solubility of Fc in water when confined within the metal–organic cage. The electrochemical data show a 300% increase in the current for the oxidation of Fc and a 570% increase in current for the reduction of electrogenerated Fc^+^ when encapsulated in Ga_4_L_6_, indicating a significant increase in the apparent solubility of Fc after encapsulation (Figure S24). These numbers are based on the current values only, and are not corrected for the adsorption of Fc on the electrode surface before addition of the metal–organic cage. While literature reports have faced challenges when attempting to accurately quantify the minute solubility of Fc in water, values ranging from 10 to 42.5 μM have been cited.^21,22,51–53^ The concentration of Ga_4_L_6_ used thus corresponds to an increase in solubility of Fc in water upon confinement of 600% to 2500% minimum. This is a lower bound estimate, as the solubility of Ga_4_L_6_ had not yet been reached, but clearly demonstrates the solubilizing effect of the presence of the metal–organic cage on Fc in water. A more precise determination of the increase in solubility would require more firm numbers on the solubility of Fc in water, which has been reported as challenging due to adsorption on electrode surfaces, trace organic solvents, and other factors. A clearer way to observe the immediate impact of confinement on the solubilization of Fc in water was thus sought. As CVs of saturated Fc solution were initially collected in a fume hood, degassing the solutions with N_2_ was required. Interestingly, during N_2_ degassing, Fc appears to volatilize out of solution at a consistent rate with further N_2_ sparging (Figure 5). The decrease in the anodic peak current for Fc as a function of sparge time follows a simple exponential decay (r^2^ = 0.999), with a pre-exponential factor of 0.999 and a half-life of 14.6 min (Figure 5, inset, blue trace).

**Figure 5:**
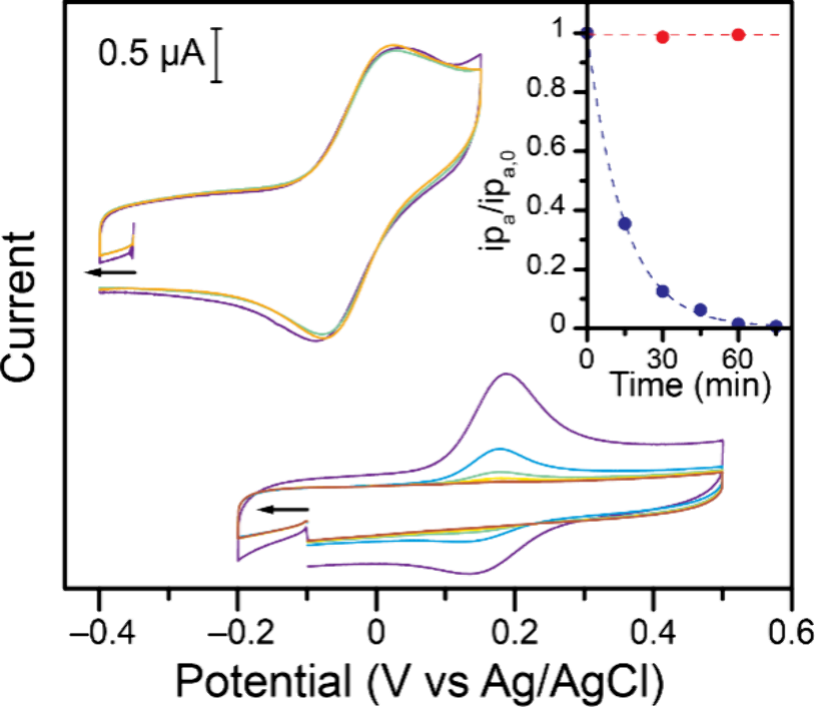
Evolution of the cyclic voltammograms of *in situ* generated Fc@Ga_4_L_6_ (top) and Fc (bottom) as a function of sparge time for 0 (purple), 15 (blue), 30 (green), 45 (yellow), 60 (orange), and 75 min (brown). The solutions were prepared and initially tested in a N_2_-filled glovebox for aqueous work, after which they were moved to a fume hood and sparged with N_2_. Data recorded in 1 M KCl in water, at 100 mV s^–1^ on a 3 mm diameter glassy carbon working electrode that remained in solution for the duration of the experiment. The inset shows the evolution of the normalized anodic peak current for Fc@Ga_4_L_6_ (red) and Fc (blue) as a function of time.

A similar behavior of loss of Fc upon sparging has been reported in ionic liquids.^54^ Fc is a ubiquitous probe in electrochemistry experiments, yet this apparent volatilization is not well established in water. To distinguish between volatilization, degradation, or nucleation of crystallites with a high kinetic barrier for redissolution, the presence of Fc in the outflow of N_2_ was tested. The outflow of N_2_ from the electrochemical cell of Fc in water was used as the inflow to sparge a second electrochemical cell, containing 0.25 M NBu_4_PF_6_ in dimethylformamide (DMF). After sparging for 110 min, CV of the DMF electrochemical cell showed a feature attributed to Fc, confirming volatilization of Fc from water as an operating pathway (Figure S25–S26).

These data confirm that Fc is typically removed from water upon sparging with N_2_. In contrast, when Fc is confined within Ga_4_L_6_, this phenomenon was not observed. The peak current intensity of confined Fc remains steady as a function of sparge time (Figure 5). This further supports the solubilizing and stabilizing effect of confinement of Fc within the Ga_4_L_6_ structure.

## Conclusion

The K_12_[Ga_4_L_6_] metal–organic cage was successfully used as a host to confine the neutral organometallic redox-active probe ferrocene in water. A combination of the known water insolubility of Fc along with the preference of the Ga_4_L_6_ host for encapsulating monocationic guests was leveraged to study electron transfer in an organometallic redox active probe under confinement. This system was able to circumvent difficulties encountered by prior reports, in which either the reduced or oxidized forms of the redox couples have low binding affinities to the hosts due to charge mismatch and/or large apertures in the host.

The encapsulation of Fc leads to its solubilization in water. The electrochemical activity of ferrocene is retained under confinement, observed as a pseudoreversible electron transfer by cyclic voltammetry. The half-wave potential is shifted negatively by 200 mV, meaning less energy is required for the oxidation of Fc. With reports of the stabilizing effect of metal–organic cages on reactive species,^55–57^ this study opens the door to the stabilization of highly active electrocatalysts. This work demonstrates the potential of confinement as a viable strategy, even for electrocatalysts, to unlock water solubility while retaining activity and reducing deleterious interactions between catalysts. The measured shift in redox potential would also help modulate the overpotential required for electrocatalysis. This work serves a foundational role in establishing the electrochemical characterization of electroactive organometallic species encapsulated within supramolecular metal–organic cages and will pave the way for advances in electrocatalysis.

## Experimental Section

### General Considerations

Reagents and solvents used were obtained from commercial sources and used without further purification unless otherwise stated. Methanol (ACS grade, VWR Chemicals BDH), potassium hydroxide (≥85%, VWR Chemicals BDH), Ga­(acac)_3_ (Strem, 99.99+%-Ga, PURATREM) and deuterium oxide (MagniSolv MilliporeSigma, 99.9% D) were used as received. Ferrocene (Alfa Aesar, 99%) was purified by sublimation prior to use. CD_3_OD (Acros Organics, 99.8% D) and dimethylformamide (DMF) were stored over 3 Å activated sieves. Potassium chloride (BDH Chemicals, 99.0–100.5%) and NBu_4_PF_6_ were recrystallized from ethanol. Reactions under an inert atmosphere were performed inside an oxygen and water-free Unilab Pro SP MBRAUN glovebox or an oxygen-free Vacuum Atmospheres Company DRI-LAB-08/85 wetbox unless specified. NMR spectra were collected on a Bruker AVanceNeo 700 MHz spectrometer equipped with a cryo-probe at 298 K or on a Bruker AVIII HD 400 MHz Nanobay spectrometer at room temperature. NMR shifts are reported as δ in ppm and referenced to the corresponding solvent residual peak.^58^ *D*_0_ values were calculated by using a Bruker pulse program with pseudo-2D sequence for diffusion measurement with stimulated echo and LED (longitudinal eddy current delay), bipolar gradient pulses for diffusion, and 2 spoil gradients at 298 K. Crystallographic data were collected at T = 100 K on a Bruker *APEX-II DUO* diffractometer equipped with a Cu microfocus source. H atoms on solvent water molecules could not be located, and disordered solvent was handled using the SQUEEZE procedure. Ligand H_4_L was synthesized according to literature procedures.^18^ All syntheses and associated workup were done in an inert atmosphere glovebox unless otherwise noted.

### Synthesis of K_12_[Ga_4_L_6_]

The K_12_[Ga_4_L_6_] host was prepared following modified literature procedures for the related system with mixed potassium and alkylammonium counterions.^18^ Ligand H_4_L (391 mg, 0.909 mmol) and Ga­(acac)_3_ (222.5 mg, 0.606 mmol) were combined in dry, degassed methanol (10 mL). KOH (499.5 mg, 8.90 mmol) in methanol was added, turning the opaque solution clear brown instantly. After stirring for a few minutes, beige-colored precipitate began to form. The solution was stirred for 5 days, after which time the precipitate was collected via filtration. Fifteen mL of methanol was added to dissolve most of the solid and the mixture was stirred for 30 s before filtration. The filtrate was collected in a clean flask and the solvent was removed under vacuum. The obtained solids were further dried under vacuum overnight, yielding 377 mg of pale-yellow powder (75% yield). ^1^H NMR characterization in agreement with prior report^18^ (400 MHz, CD_3_OD) δ: 8.06 (d, 12H, Ar*H*), 7.80 (d, 12H, Ar*H*), 7.27 (d, 12H, cat*H*), 7.00 (t, 12H, Ar*H*), 6.68 (d, 12H, cat*H*), 6.40 (t, 12H, cat*H*).

### Synthesis of Fc@Ga_4_L_6_

Ferrocene (8.8 mg, 0.047 mmol) was added to a Schlenk flask. Separately, K_12_[Ga_4_L_6_] (100 mg, 0.030 mmol, Fc is in excess) was dissolved in 6 mL of degassed water and then transferred to the Schlenk flask. The flask was sealed, brought out of the inert atmosphere wetbox, and placed in an ultrasonic bath for 25 min. The flask was brought back into an inert atmosphere wetbox and the solution was filtered, and the clear golden yellow filtrate was collected into a clean Schlenk flask. The solvent was removed under vacuum, in a 30 °C water bath to aid solvent removal. The obtained golden-colored flakes were further dried under vacuum overnight, yielding 84 mg (79.5%). ^1^H NMR (700 MHz, D_2_O) δ: 7.90 (d, J = 7.8 Hz, 12H, Ar*H*), 7.85 (d, J = 8.5 Hz, 12H, Ar*H*), 7.34 (d, J = 8.4 Hz, 12H, cat*H*), 7.10 (t, J = 8.2 Hz, 12H, Ar*H*), 6.77 (d, J = 7.5 Hz, 12H, cat*H*), 6.61 (t, J = 7.8 Hz, 12H, cat*H*), 0.76 (s, 10H, encapsulated Fc). ^13^C NMR (700 MHz, D_2_O) δ: 169.66, 158.53, 154.78, 133.04, 126.90, 125.82, 119.16, 117.85, 115.43, 114.70, 114.66, 114.37, 64.15. XRD structure deposited under CCDC2473738.

### Electrochemical Measurements

The IUPAC convention was used to report currents and plot cyclic voltammetry data. Data was recorded using a BioLogic SP 300 or BioLogic BP 300 potentiostat. The effects of ohmic drop were mitigated by using the built-in ZIR compensation feature of the potentiostats. Cyclic voltammograms were collected using a three-electrode setup with a glassy carbon working electrode (3 mm diameter, CH Instruments), platinum counter electrode (2 mm diameter, CH Instruments), and a Ag/AgCl 1.00 M KCl reference electrode (CH Instruments) which was stored in 1 M KCl. The electrochemical cell used was a 20 mL borosilicate glass vial with a custom machined PTFE cap to accommodate passage of the electrodes and sparge tubes. Working and counter electrodes were polished using type N Alpha alumina powder (0.05 μm, Electron Microscopy Sciences) on separate microcloth polishing pads (CH Instruments). Electrodes were pretreated by cycling between the upper and lower potential boundaries of the electrochemical window of the solvent in a solution containing only the electrolyte. Measurements of K_12_[Ga_4_L_6_] and Fc@Ga_4_L_6_ were taken in degassed solutions with 1.0 M recrystallized KCl, inside of a nitrogen-filled wetbox or in a fume hood with a stream of N_2_ above the solution. Analyte was typically added to 10 mL of electrolyte solution to give the desired concentration. The saturated Fc solution was prepared by adding excess Fc (1.2 mg) to 15 mL of 1.0 M KCl in water. The suspension was then sonicated for 1 h before it was allowed to stand for 24 h. The suspension was then filtered to remove remaining solids and was added directly to an electrochemical cell. During the collection of scan rate variation CVs of saturated Fc, the electrodes were rinsed in pure CH_3_CN, then rinsed in water, and dried under N_2_ between each scan.

## Supplementary Material



## Notes

Additional NMR and X-ray characterization and electrochemical data and analysis (PDF)
