# Supplementary material for: Imparting Water Solubility and Aqueous Electrochemical Activity to Ferrocene upon Confinement
Source: Inorg Chem. 2026 Apr 29;65(18):10362–9. doi: 10.1021/acs.inorgchem.6c01327 (PMC13169389; doi:10.1021/acs.inorgchem.6c01327)
Supplement: Supplementary file 1 [file ic6c01327_si_001.pdf]

Supporting Information

Imparting water solubility and aqueous electrochemical activity to ferrocene upon confinement

*Ryan J. Bujol,[‡] Nathan H. Mitchell,[‡] Siddhiaratchige D. M. Siddhiaratchi, Thomas K. Weldeghiorghis, Frank R. Fronczek, and Noémie Elgrishi**

Department of Chemistry, Louisiana State University, Baton Rouge,

Louisiana, 70803, United States

[‡] These authors contributed equally to this work

*Correspondence to: noemie@lsu.edu

Index *Page*

**NMR Spectroscopy Data**		
^{1}H NMR Spectrum of a saturated Fc solution	Figure S1	S3
^{1}H NMR Characterization of Fc@Ga_4L_6	Figure S2	S4
^{13}C NMR Characterization of Fc@Ga_4L_6	Figure S3	S5
HSQC NMR Characterization of Fc@Ga_4L_6	Figure S4	S5
NOESY NMR Characterization of Fc@Ga_4L_6	Figure S5	S6
**Single Crystal X-ray Diffraction Data**	Figure S6	S7
**Diffusion-Ordered Spectroscopy (DOSY)**		
Fc@Ga_4L_6 in D_2O	Figure S7	S8
Fc@Ga_4L_6 in D_2O with 1 M KCl	Figure S8	S8
$K_{12}[Ga_4L_6]$ in D_2O	Figure S9	S9
$K_{12}[Ga_4L_6]$ in D_2O with 1 M KCl	Figure S10	S9
$K_{12}[Ga_4L_6]$ in CD_3OD	Figure S11	S10
Fc in CD_3OD	Figure S12	S10
Fc@Ga_4L_6 in CD_3OD	Figure S13	S11
**Electrochemical Data**		
Methodology to Determine D^0 and k^0 Values		S12
CV of $K_{12}[Ga_4L_6]$	Figure S14	S13
Representative CVs of Fc@Ga_4L_6 at various scan rates	Figure S15	S13
Analysis of Figure S15	Figure S16-S18	S14
Trumpet Plot estimation of average k_0 for Fc@Ga_4L_6	Figure S19	S16
CVs of Fc in water and corresponding analysis	Figure S20-S22	S17
CVs of Fc@Ga_4L_6 as synthesized vs in situ generated	Figure S23	S19
Increase in Fc signal in CV upon formation of Fc@Ga_4L_6	Figure S24	S20
Transfer of Fc through sparging	Figure S25-S26	S21
**References**		S22

NMR Spectroscopy Data

¹H NMR of a saturated solution of Fc in D_2O

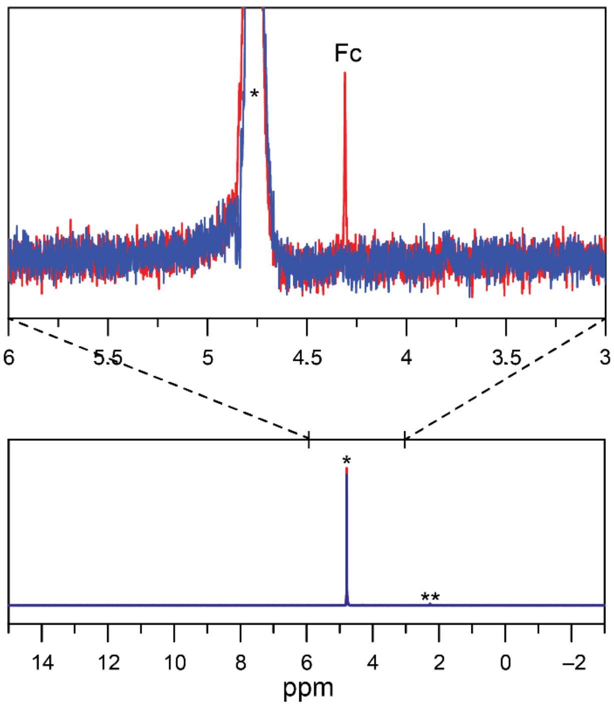

Figure S1. ^{1}H NMR of a saturated solution of Fc in D_2O (red) and of the blank NMR solvent (blue). Data collected at room temperature on a 400 MHz spectrometer. *: Solvent residual peak at 4.79 ppm, **: trace acetone at 2.2 ppm, Fc: peak attributed to Ferrocene at 4.3 ppm.

NMR characterization of Fc@Ga_4L_6 in D_2O

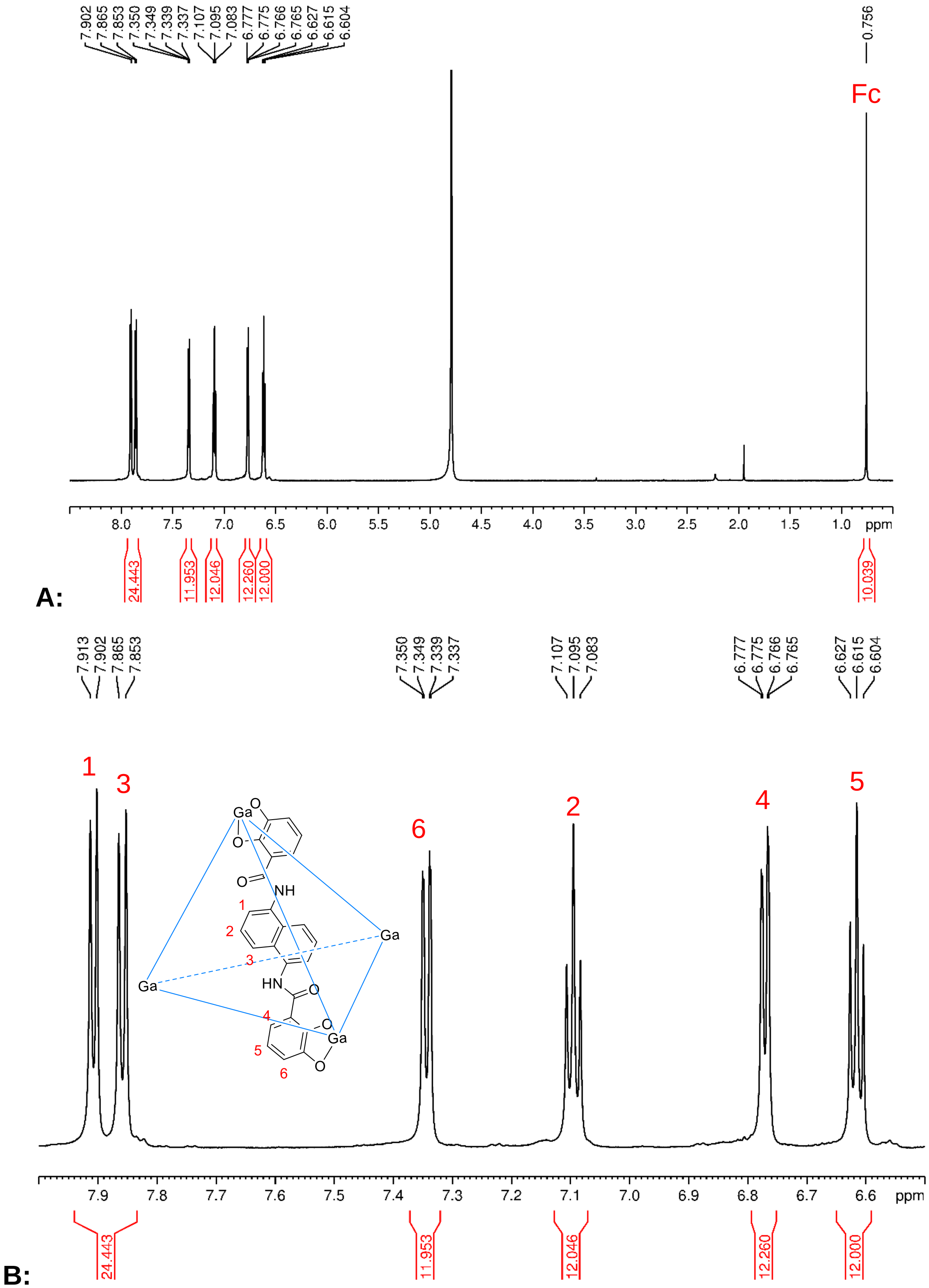

Figure S2. ^1H NMR profile of Fc@Ga_4L_6 in D_2O (residual peak at 4.79 ppm) collected at 298 K on a 700 MHz spectrometer. A: full spectrum with the encapsulated Fc peak observed at 0.76 ppm. B: aromatic region with assignments.

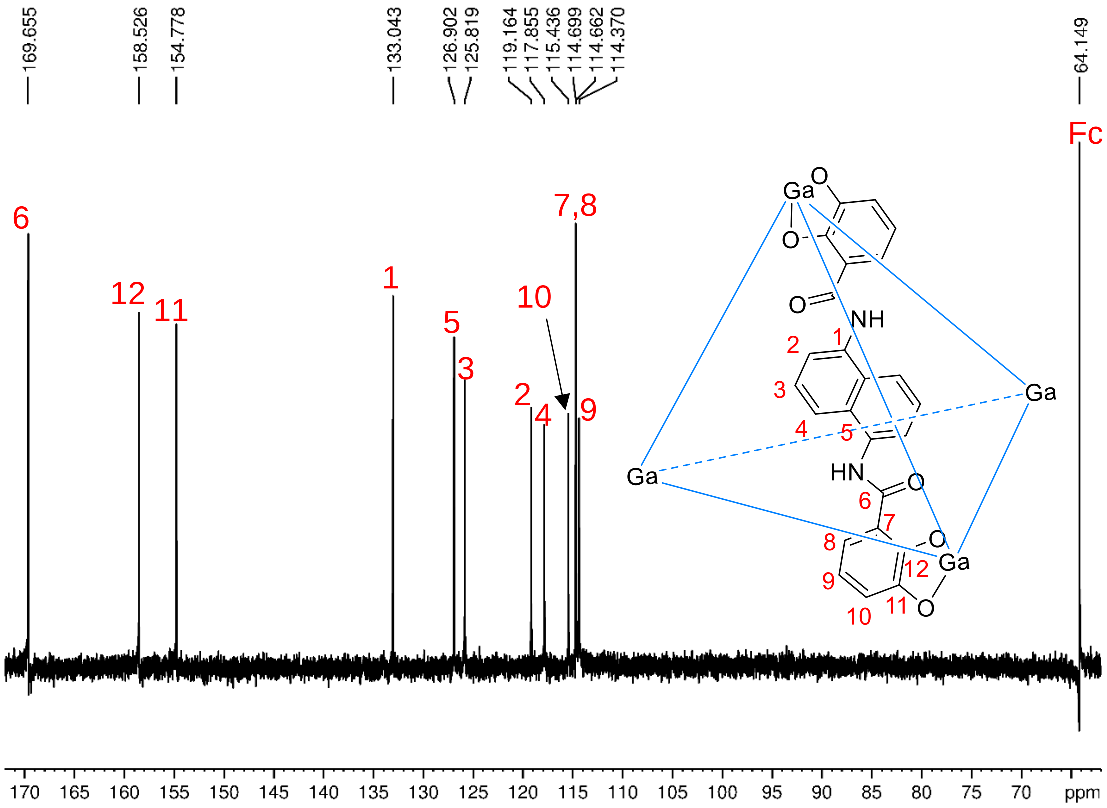

Figure S3. ^{13}C NMR profile of Fc@Ga_4L_6 in D_2O collected at 298 K on a 700 MHz spectrometer.

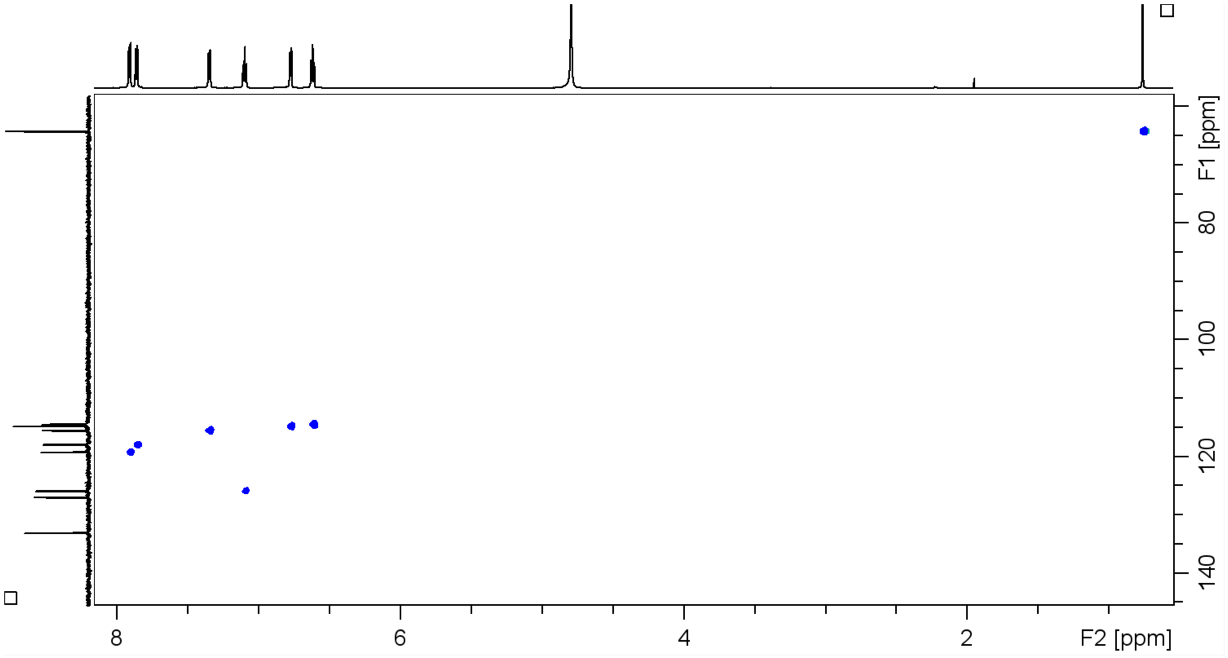

Figure S4. 2D HSQC NMR of Fc@Ga_4L_6 in D_2O collected on a 700 MHz spectrometer at 298 K.

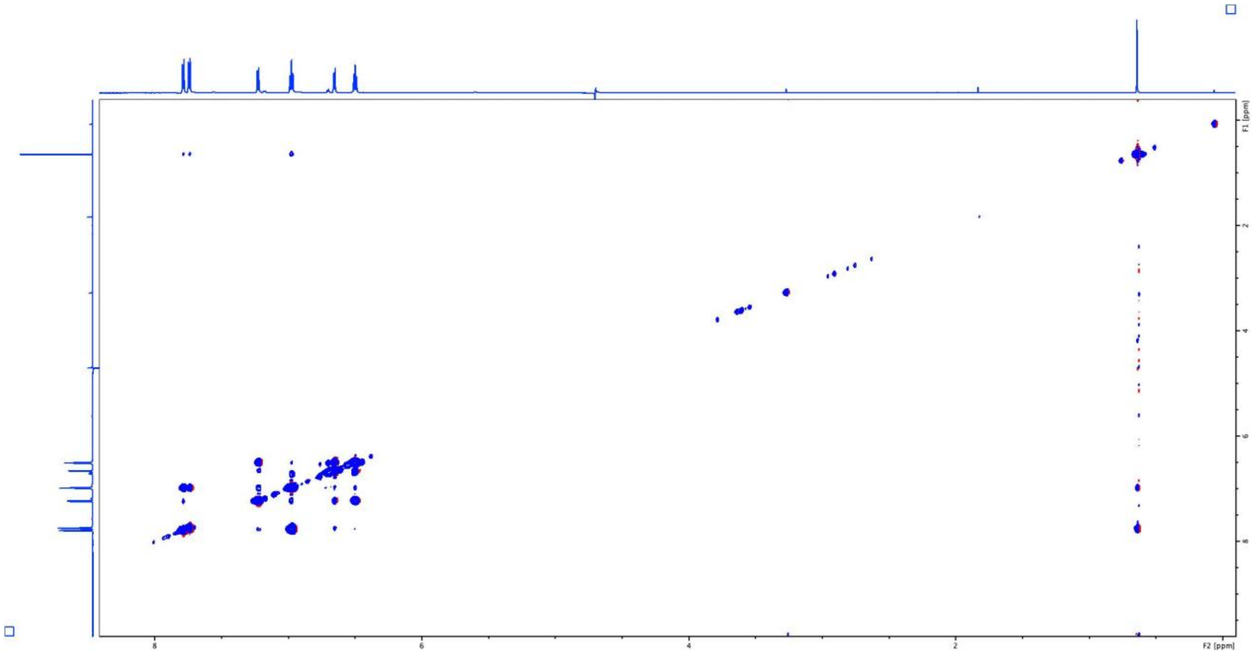

Figure S5. 2D NOESY spectrum collected on a 700 MHz instrument at 298 K for Fc@Ga_4L_6 in D_2O and mixing time was 600 ms.

Single Crystal X-ray Diffraction Data

Crystals suitable for X-ray diffraction were grown by slow evaporation of an aqueous solution of Fc@Ga_4L_6. Due to oxygen sensitivity, this was conducted within an air-free wetbox. Small vials of aqueous Fc@Ga_4L_6 solution were placed in a large media jar containing Drierite™. The large media jar was sealed, and the system was left to slowly crystallize. Crystal quality was poor, but sufficient to establish the connectivity. Crystal Data: monoclinic space group Cc with Z = 8, a = 16.6979(5), b = 32.5849(10), c = 64.9960(19) Å, β = 92.478(2)°, V = 35331.2(18) Å^3 at T = 100K. Data were collected on a Bruker Kappa Apex-II DUO diffractometer to θ_{max} = 66.95° with CuKα radiation from a microfocus source. H atoms were placed in idealized positions where possible, but those on water molecules could not be located. Disordered solvent was removed using the SQUEEZE procedure. The structure was refined as a 2-component inversion twin, with Flack parameter 0.152(12). 186,665 measured reflections, 61,291 unique, 45,055 observed with I>2σ(I). R = 0.121 for 4367 refined parameters. CCDC2473738.

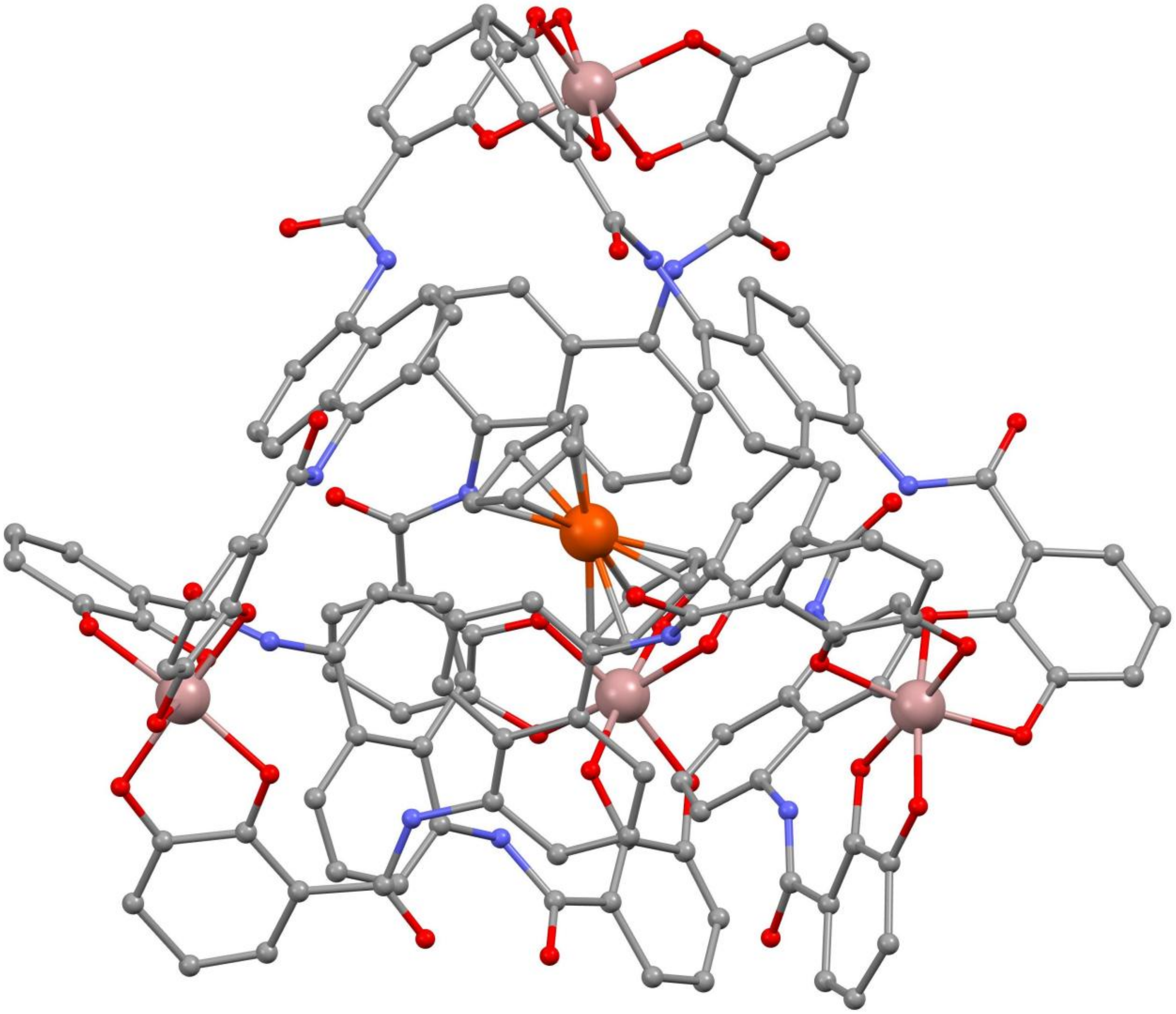

Figure S6. One of the two crystallographically independent complexes. Ellipsoids and H atoms are not shown. Atoms: carbon in grey, oxygen in red, nitrogen in blue, gallium in pink, and iron in orange.

Diffusion-Ordered Spectroscopy (DOSY)

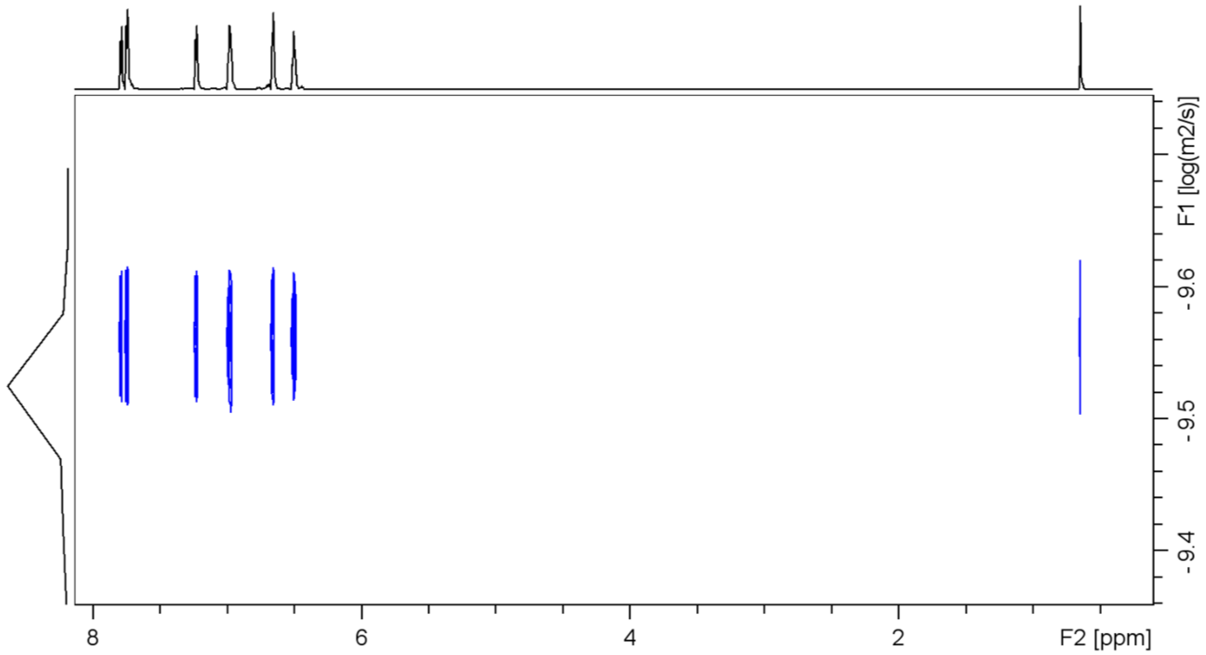

Figure S7. DOSY spectrum of $Fc@Ga_4L_6$ in D_2O on a 700 MHz instrument at 298 K. For the Ga_4L_6 peaks, an average D_0 value of 2.4 × 10^{-6} cm^2 s^{-1} was calculated, and of 2.4 × 10^{-6} cm^2 s^{-1} for the Fc peak.

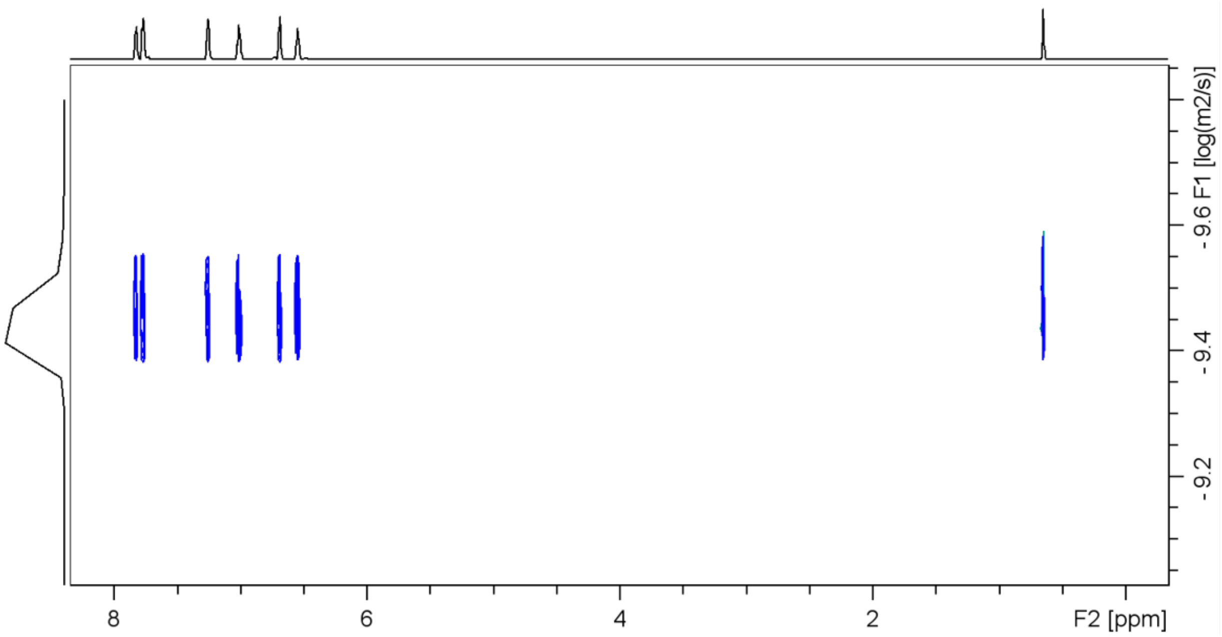

Figure S8. DOSY spectrum of $Fc@Ga_4L_6$ in 1 M KCl in D_2O on a 700 MHz instrument at 298 K. An average D_0 value of 3.0 × 10^{-6} cm^2 s^{-1} was calculated for the Ga_4L_6 peaks, and of 3.0 × 10^{-6} cm^2 s^{-1} for the Fc peak.

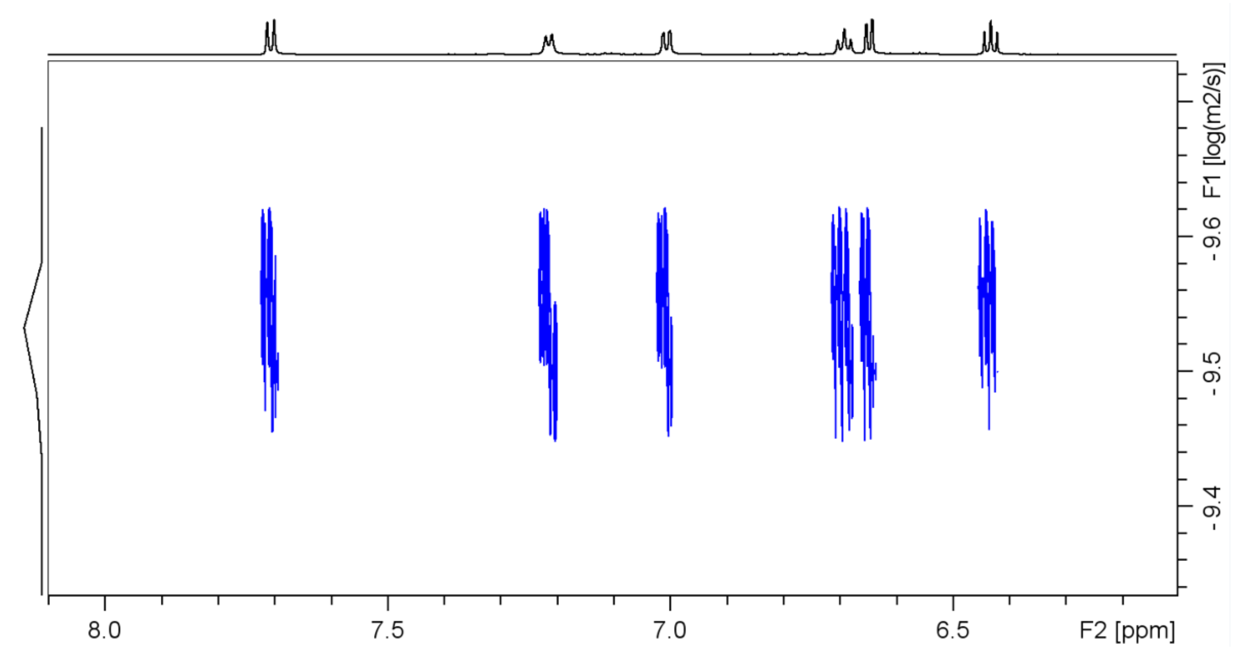

Figure S9. DOSY spectrum of $K_{12}[Ga_4L_6]$ in D_2O on a 700 MHz instrument at 298 K. An average D_0 value of 2.4×10^{-6} cm^2 s^{-1} was calculated.

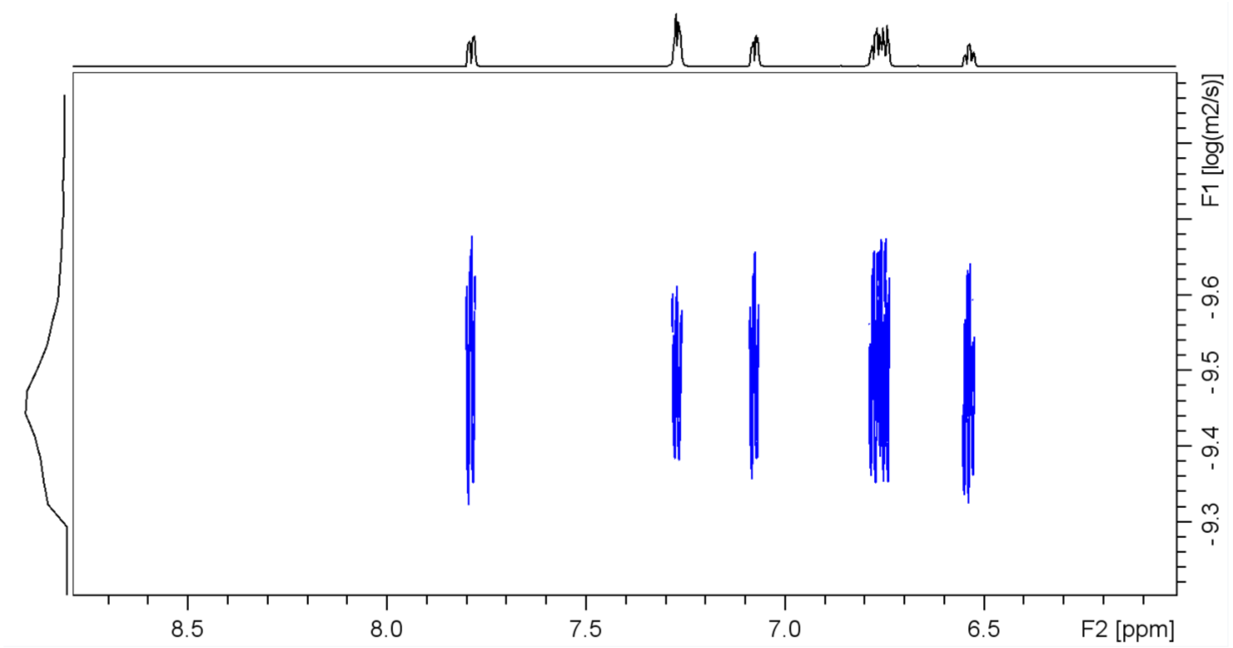

Figure S10. DOSY spectrum of $K_{12}[Ga_4L_6]$ in 1M KCl in D_2O on a 700 MHz instrument at 298 K. An average D_0 value of 3.6×10^{-6} cm^2 s^{-1} was calculated.

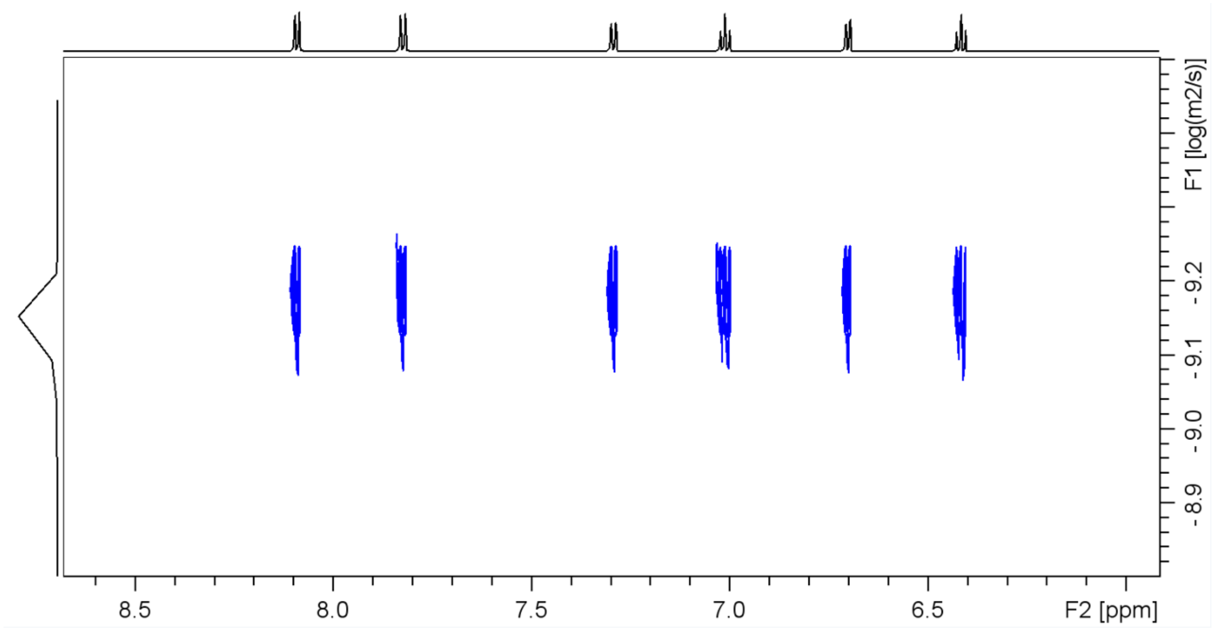

Figure S11. DOSY spectrum of $K_{12}[Ga_4L_6]$ in CD_3OD on a 700 MHz instrument at 298 K. An average value of $D_0 = 5.6 \times 10^{-6}$ $cm^2\,s^{-1}$ was calculated.

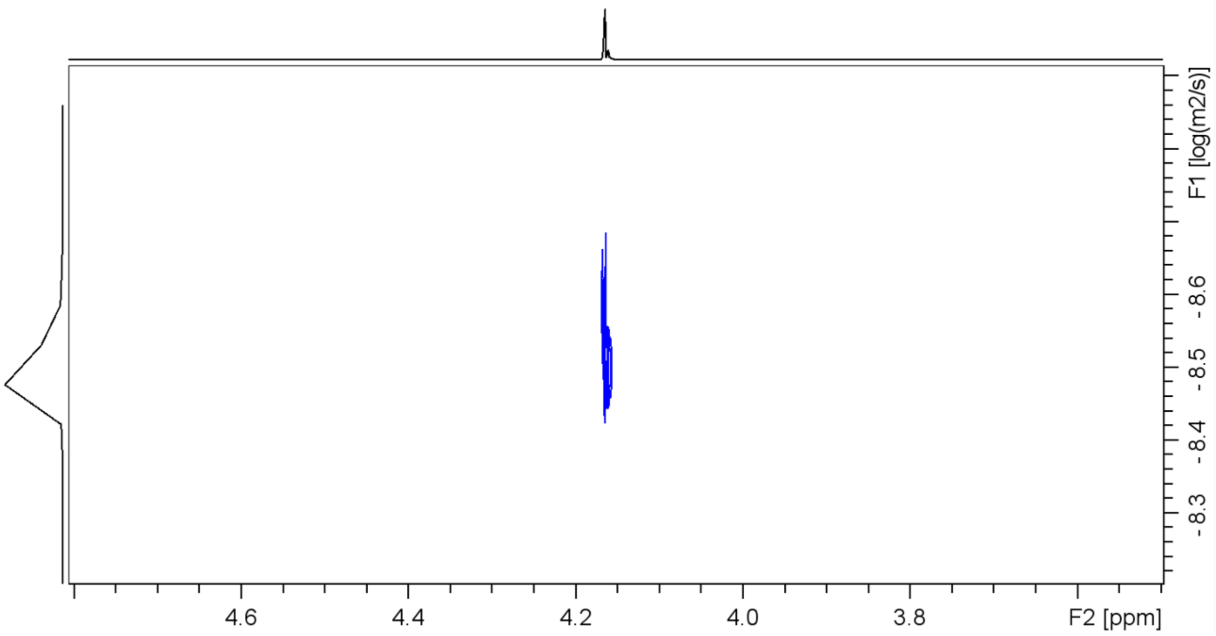

Figure S12. DOSY spectrum of Fc in CD_3OD on a 700 MHz instrument at 298 K. A D_0 value of 2.6×10^{-5} $cm^2\,s^{-1}$ was calculated.

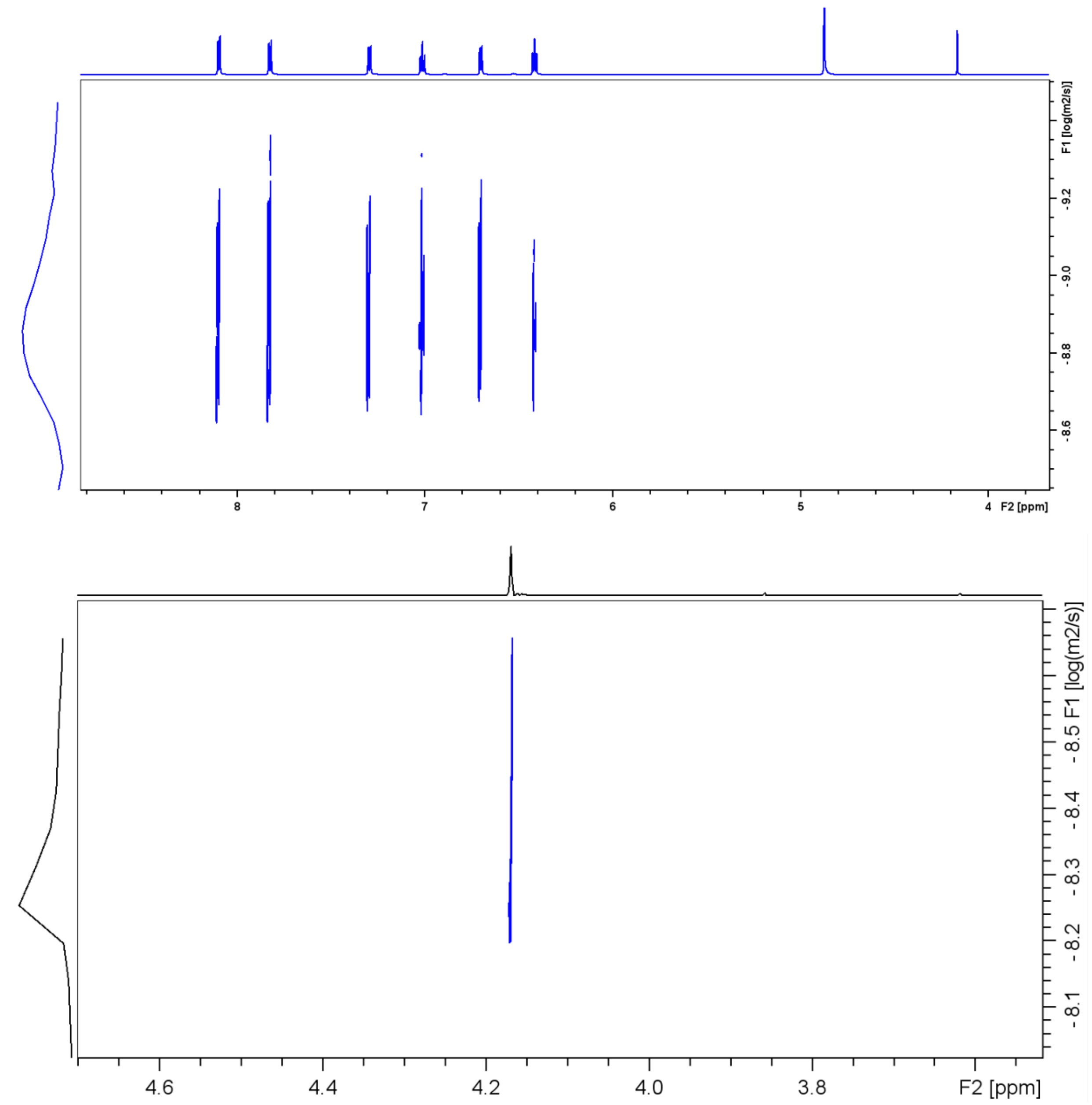

Figure S13. DOSY spectrum of Fc@Ga_4L_6 in CD_3OD optimized for the Ga_4L_6 peaks (top) and for the Fc peak (bottom). Data collected on a 700 MHz instrument at 298 K. An average D_0 value of 5.8×10^{-6} cm^2 s^{-1} was calculated for the Ga_4L_6 aromatic peaks, and of 2.9×10^{-5} cm^2 s^{-1} for the Fc peak.

Electrochemical Data

Methodology to Determine D_0 and k_0 Values

More details for the calculations of Diffusion coefficients (D_0) and heterogeneous electron transfer rate constants (k_0) can be found in a previous report.[1]

Briefly, Faradaic peak currents are plotted against the square root of the scan rate using scan rate variation data. D_0 can then be calculated from the resulting slope using the Randles-Sevcik equation (eq. *S*1):

$$i_p = 0.4463FAC_0\left(\frac{nF\upsilon D_0}{RT}\right)^{\frac{1}{2}} \qquad (S1)$$

Where i_p is peak current in A, *F* is Faraday's constant, *R* is the ideal gas constant in J mol^{-1} K^{-1}, *T* is temperature in K, *n* is the number of electrons in the redox reaction, *A* is the surface area of the electrode in cm^2, D_0 is the diffusion coefficient in cm^2 s^{-1}, C_0 is concentration in mol cm^{-3}, and υ is the scan rate in V s^{-1}.[2] In the triplicate analysis in Figure 4 of the main text, the Faradaic peak current was normalized by the concentration C_0 to account for variations in concentrations between datasets.

The Nicholson method was used to determine k_0 values.[3–5] Briefly, peak-to-peak separations ΔE_p (in mV) were determined at each scan rate and used to calculate the Nicholson parameter Ψ using equation *S*2:

$$\Psi = \frac{-0.6288 + 0.0021\Delta E_p}{1 - 0.017\Delta \mathrm{E}_p} \qquad (S2)$$

The Nicholson parameter Ψ is then plotted as a function of the inverse square root of the scan rate (in V s^{-1}) and k_0 values are determined from the slope of the resulting graph which follows the equation *S*3:

$$\Psi = k_0\left(\frac{\pi F\upsilon D}{RT}\right)^{-\frac{1}{2}} \qquad (S3)$$

Where k_0 is the electron transfer rate constant in cm s^{-1} *D* is the average of the diffusion coefficients for the reduced and oxidized species of the redox couple in cm^2 s^{-1}, F is Faraday's constant, υ is the scan rate in V s^{-1}, R is the ideal gas constant in J mol^{-1} K^{-1}, and T is temperature in K.[4,6]

Another estimate of the electron transfer rate constant k_0 was provided by a Trumpet Plot Analysis, described in detail elsewhere.[7] Briefly, the position of the anodic and cathodic peak potentials relative to the $E_{1/2}$ at each scan rate is plotted as a function of the log of the scan rate and fitted to a working curve for a compound with D = 1 × 10^{-5} cm^2 s^{-1} and *k* = 1 cm s^{-1}. The offset needed to the working curve to fit the data, along with the value of the diffusion coefficient of the analyte in these conditions, is used to determine k_0.

CV of Ga_4L_6

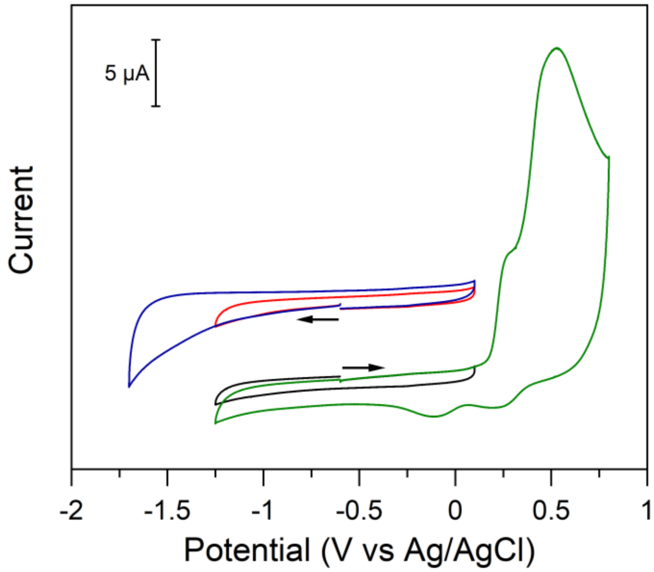

Figure S14. Cyclic voltammograms obtained for a sample containing 0.1 mM of $K_{12}[Ga_4L_6]$ in 3.0 mL of H_2O with 1 M KCl as supporting electrolyte. The CVs were recorded at 100 mV s^{-1}.

Representative CVs of Fc@Ga_4L_6 at various scan rates

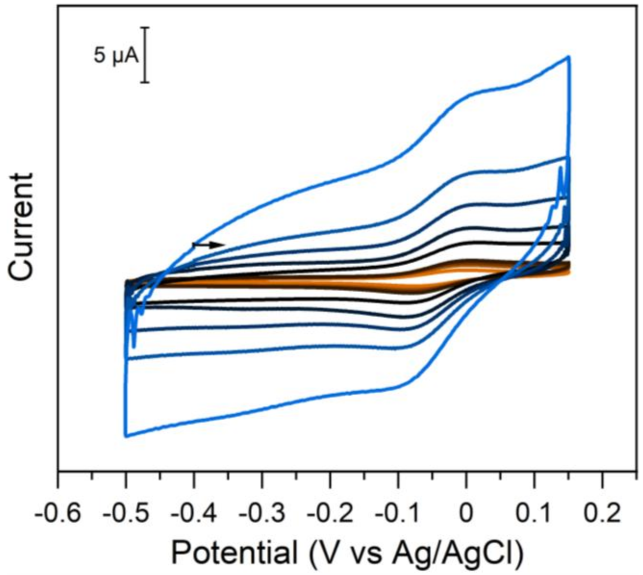

Figure S15. Cyclic voltammograms obtained for a sample containing 0.25 mM Fc@Ga_4L_6 in H_2O with 1 M KCl as supporting electrolyte. Second cycles showed. Data recorded on freshly polished 3 mm diameter glassy carbon working electrodes at scan rates of, from orange to blue: 0.025, 0.050, 0.075, 0.10, 0.25, 0.50, 0.75, 1.00, and 2.00 V $s^{-1.}$

Determination of D_0 for the representative CVs of $Fc@Ga_4L_6$

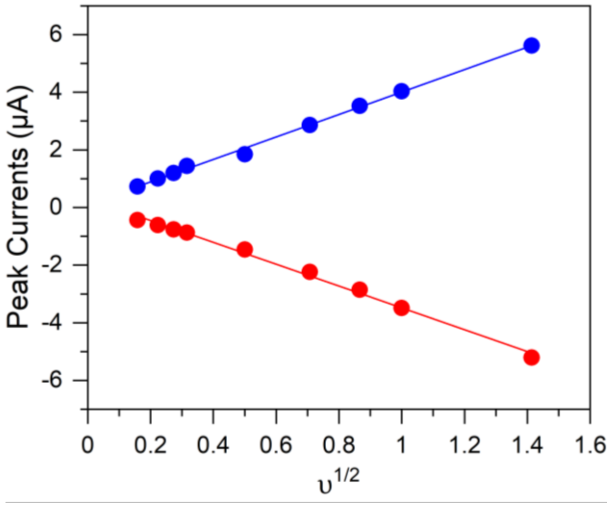

Figure S16. Evolution of the Faradaic anodic (blue) and cathodic (red) peak currents as a function of the square root of the scan rate for CVs of 0.25 mM of $Fc@Ga_4L_6$ in water with 1M KCl electrolyte from Figure S15. The slopes of the linear fits (oxidation: 3.897 μA $V^{-1/2}$ $s^{1/2}$ with r^2 = 0.997; Reduction: −3.790 μA $V^{-1/2}$ $s^{1/2}$ with r^2 = 0.995) allow for the determination of the diffusion coefficient following the Randles–Ševčík equation. A D_0 value of 6.49 × 10^{-7} cm^2 s^{-1} was obtained for the oxidation, and 6.14 × 10^{-7} cm^2 s^{-1} for the reduction for this specific data set.

Nicholson method determination of k_0 for the representative CVs of $Fc@Ga_4L_6$

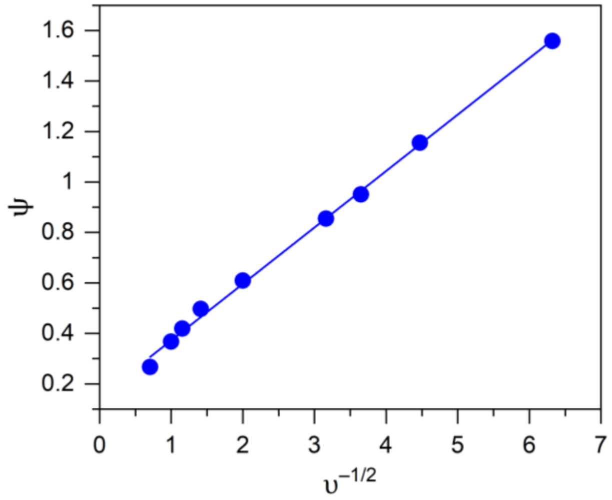

Figure S17. Plot of Ψ vs $\upsilon^{-1/2}$ following the Nicholson method for CVs of 0.25 mM of $Fc@Ga_4L_6$ in water with 1M KCl electrolyte from Figure S15. The slope of the linear fit (0.224 $V^{-1/2}$ $s^{1/2}$ with r^2 = 0.998) was used, along with the average value of D_0 = 6.31 × 10^{-7} cm^2 s^{-1}, to determine the heterogeneous electron transfer rate constant of k_0 = 0.00198 cm s^{-1}.

Estimation of k_0 for the representative CVs of Fc@Ga_4L_6 using the Trumpet Plot method

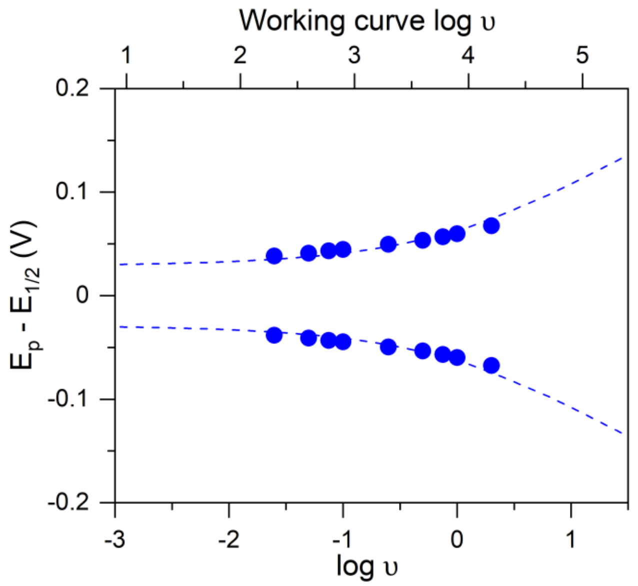

Figure S18. Trumpet plot for CVs of 0.25 mM of Fc@Ga_4L_6 in water with 1M KCl electrolyte from Figure S15 (blue circles, bottom x-axis). The data is fitted to a working curve (dashed lines, top x-axis)[7] corresponding to D = 1×10^{-5} cm^2 s^{-1} and k = 1 cm s^{-1}.The offset (Δ = –3.9) between the data and the x-axis of the best fitting working curve, along with the value of $D_0 = 6.31 \times 10^{-7}$ cm^2 s^{-1} is used to determine an estimation of the heterogeneous electron transfer rate constant of k_0 = 0.00282 cm s^{-1}.

Average estimation of k_0 for Fc@Ga_4L_6 using the Trumpet Plot method

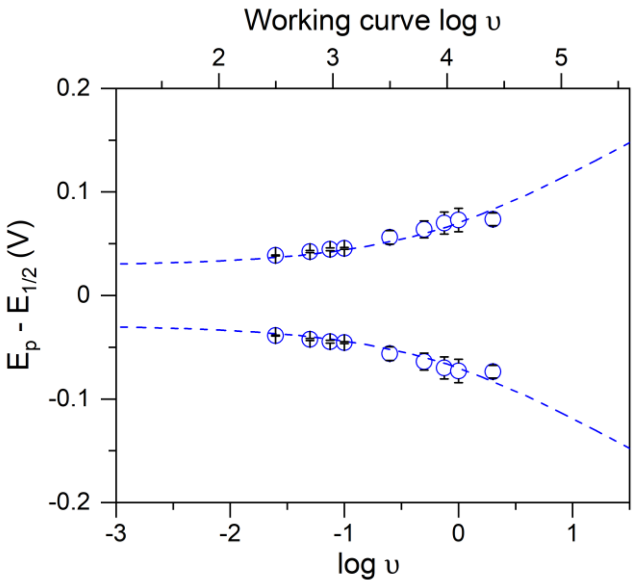

Figure S19. Trumpet plot analysis[7] using average peak potential values for triplicate scan rate dependence experiments on 0.25 mM Fc@Ga_4L_6 in 1 M KCl in water. An estimation of the heterogeneous electron transfer rate constant of k_0 = 0.00224 cm s^{-1} is obtained (Δ = –4.1; D_0 = 6.34 × 10^{-7} cm^2 s^{-1}).

CVs of Fc in water

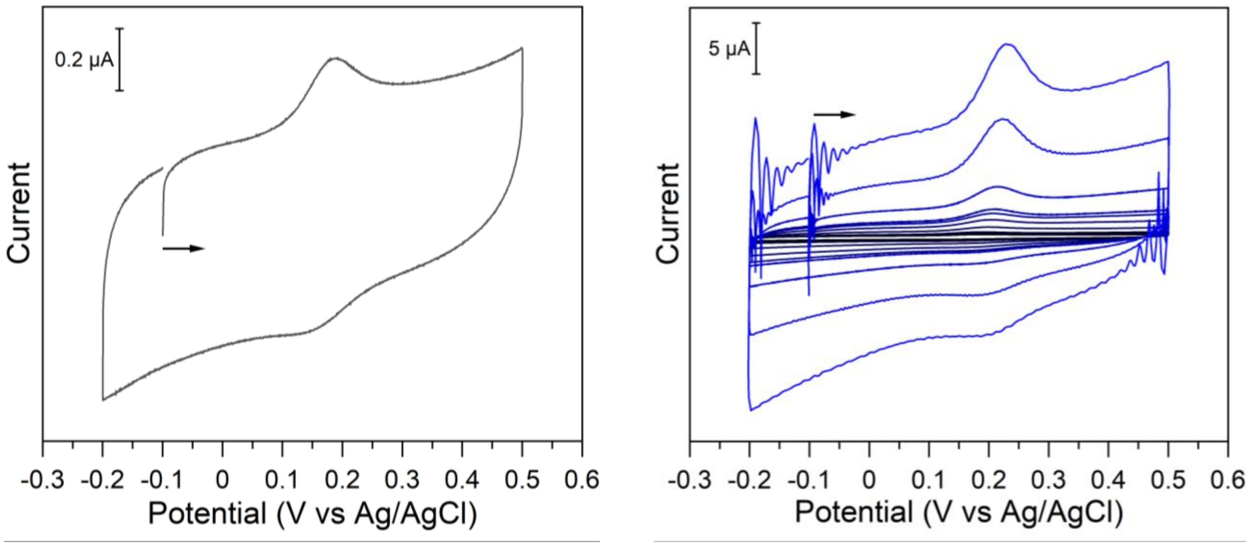

Figure S20. CVs of Fc in 1 M KCl in water. Fc solid was added to water with 1 M KCl and the solution was stirred, then filtered, and only then added to an electrochemical cell. The CVs were collected using a 3 mm diameter glassy carbon working electrode that was rinsed with acetonitrile and dried between each scan. Example CV at collected at 100 mV s^{-1} (left) and at varying scan rates of 0.050, 0.075, 0.100, 0.250, 0.500, 0.750, 1.00, 2.00, 5.00, and 10.0 V s^{-1} (right, from black to blue).

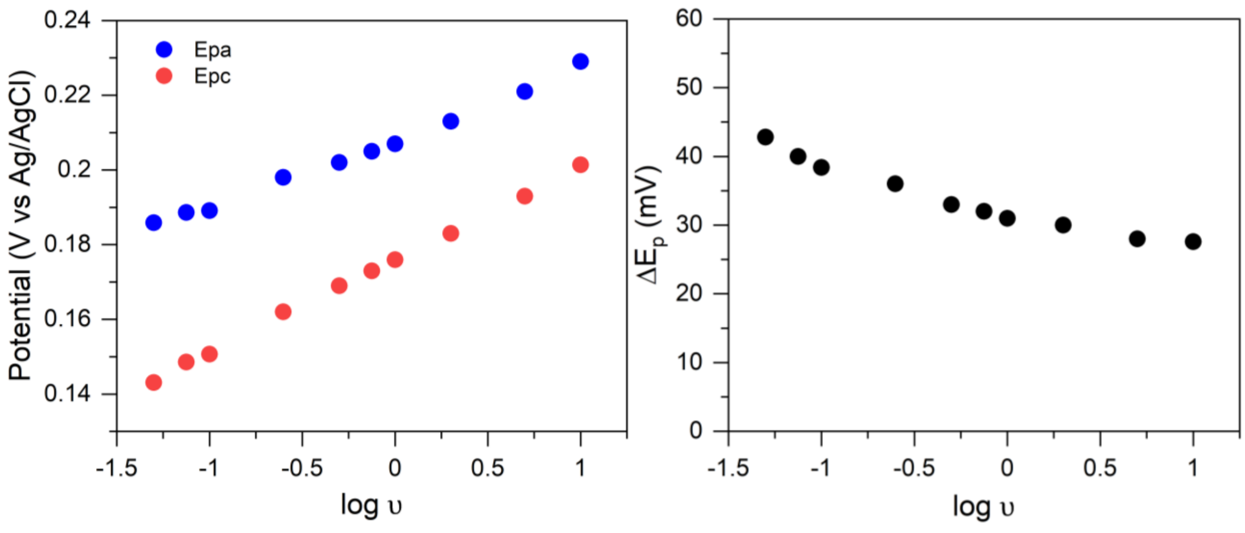

Figure S21. Evolution of E_{pc} and E_{pa} (left) and of ΔE_p (right) as a function of the log of the scan rate for a solution of saturated Fc in 1 M KCl in H_2O (CV data in Figure S20).

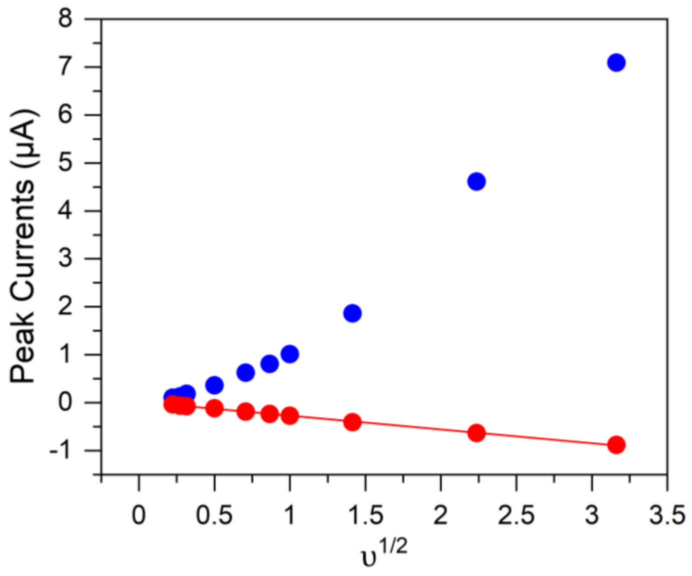

Figure S22. Plot of the anodic (blue) and cathodic (red) peak currents as a function of the square root of the scan rate (CV data from Figure S20). While the anodic peak currents do not trend linearly with $\upsilon^{1/2}$, the linear fit of the cathodic peak currents has a slope of −0.288 μA $V^{-1/2}$ $s^{1/2}$ (r^2 = 0.999). The exact concentration of Fc in the solution is unknown, given the solubility challenges and known adsorption onto the electrode surface.

CV comparison of Fc@Ga_4L_6 as synthesized vs *in situ* generated

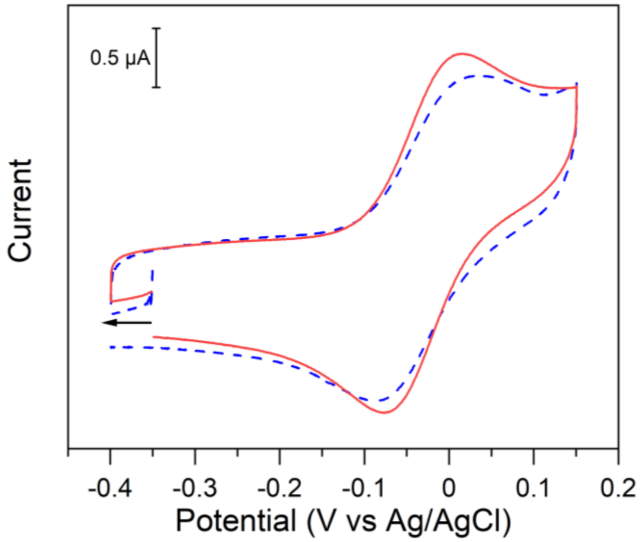

Figure S23. CVs of *in situ* generated 0.25 mM Fc@Ga_4L_6 in 1 M KCl (blue) compared to an authentic sample of 0.25 mM Fc@Ga_4L_6 in 1 M KCl (red). The *in situ* generated Fc@Ga_4L_6 was prepared by adding excess Fc to Ga_4L_6 in 2 mL of H_2O and stirring for 2 hours. The solution was not filtered and diluted with 1 M KCl to give 0.25 mM Fc@Ga_4L_6 in water based on the starting mass of Ga_4L_6. CVs were collected using freshly polished 3 mm diameter glassy carbon working electrodes at 100 mV s^{-1}.

Increase in Fc signal in CV upon formation of Fc@Ga_4L_6

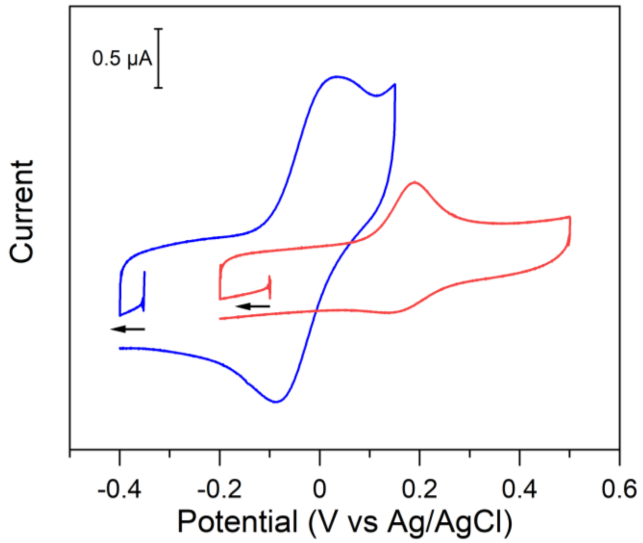

Figure S24. CV of *in situ* generated 0.25 mM Fc@Ga_4L_6 (blue) and of a saturated Fc solution (red) in 1 M KCl at 100 mV s^{-1}. CVs recorded on a 3 mm diameter glassy carbon working electrode. The saturated Fc solution was not filtered before recording the CV and accumulation on the electrode surface is likely in these conditions. Ga_4L_6 was added to that same solution and Fc@Ga_4L_6 was generated *in situ*: the CV is recorded in the presence of excess Fc.

Transfer of Fc through sparging

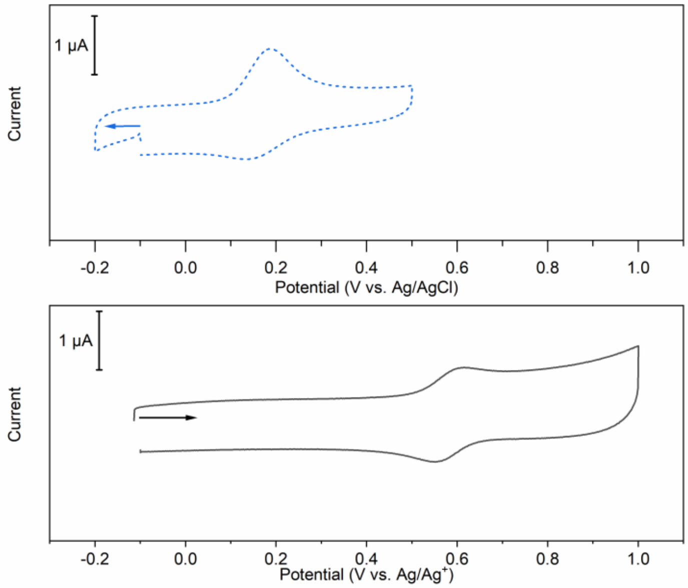

Figure S25. CV of a saturated Fc solution in 1 M KCl (top, blue). The solution was then sparged with $N_{2(g)}$, and the PTFE tube gas outlet of the cell was connected to sparge a solution of 0.25 M NBu_4PF_6 in DMF. After 110 minutes of sparging, CVs were collected in the DMF cell, which had a Ag wire in the electrolyte as a pseudo-reference electrode, and a freshly polished working electrode pretreated in the DMF electrolyte. The CV (bottom, black) shows a reversible redox couple at a potential which matches that of the Fc^+/Fc in these conditions (see figure S26). CVs recorded at 100 mV s^{-1} on a 3 mm diameter glassy carbon working electrode.

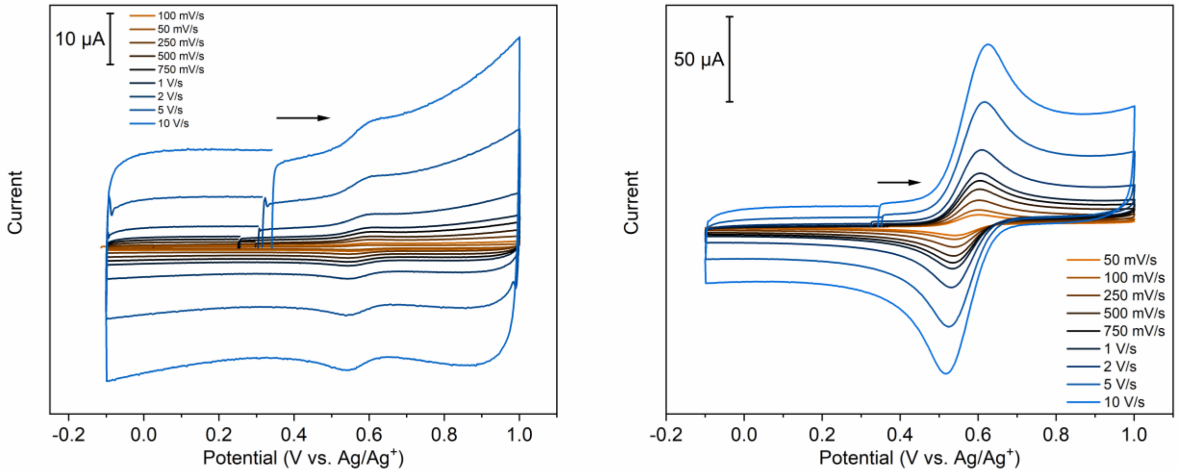

Figure S26. CVs in 0.25 M NBu_4PF_6 in DMF at variable scan rates, collected on a 3 mm glassy carbon working electrode. Left: CVs of Fc sparged transferred from saturated Fc in 1 M KCl. Right: CVs of 1.00 mM Fc, with an identical $E_{1/2}$.

Randles-Sevcik analysis of both datasets determined the concentration of Fc in the sparge-transferred solution to be 0.047 mM.

References

(1) Mitchell, N. H.; Elgrishi, N. Investigation of Iron(III) Tetraphenylporphyrin as a Redox Flow Battery Anolyte: Unexpected Side Reactivity with the Electrolyte. *J. Phys. Chem. C* **2023**, *127* (23), 10938–10946. https://doi.org/10.1021/acs.jpcc.3c01763.

(2) Elgrishi, N.; Rountree, K. J.; McCarthy, B. D.; Rountree, E. S.; Eisenhart, T. T.; Dempsey, J. L. A Practical Beginner’s Guide to Cyclic Voltammetry. *J. Chem. Educ.* **2018**, *95* (2), 197–206. https://doi.org/10.1021/acs.jchemed.7b00361.

(3) Nicholson, R. S. Theory and Application of Cyclic Voltammetry for Measurement of Electrode Reaction Kinetics. *Anal. Chem.* **1965**, *37* (11), 1351–1355. https://doi.org/10.1021/ac60230a016.

(4) Wang, H.; Sayed, S. Y.; Luber, E. J.; Olsen, B. C.; Shirurkar, S. M.; Venkatakrishnan, S.; Tefashe, U. M.; Farquhar, A. K.; Smotkin, E. S.; McCreery, R. L.; Buriak, J. M. Redox Flow Batteries: How to Determine Electrochemical Kinetic Parameters. *ACS Nano* **2020**, *14* (3), 2575–2584. https://doi.org/10.1021/acsnano.0c01281.

(5) Lavagnini, I.; Antiochia, R.; Magno, F. An Extended Method for the Practical Evaluation of the Standard Rate Constant from Cyclic Voltammetric Data. *Electroanalysis* **2004**, *16* (6), 505–506. https://doi.org/10.1002/elan.200302851.

(6) Berben, L. A.; Arnold, A.; Dougherty, R. J.; Carr, C. R.; Reynolds, L. C.; Fettinger, J. C.; Augustin, A. A Stable Organo-Aluminum Analyte Enables Multielectron Storage for a Nonaqueous Redox Flow Battery. *J. Phys. Chem. Lett.* **2020**, *11* (19), 8202–8207. https://doi.org/10.1021/acs.jpclett.0c01761.

(7) Rountree, E. S.; Martin, D. J.; McCarthy, B. D.; Dempsey, J. L. Linear Free Energy Relationships in the Hydrogen Evolution Reaction: Kinetic Analysis of a Cobaloxime Catalyst. *ACS Catal.* **2016**, *6* (5), 3326–3335. https://doi.org/10.1021/acscatal.6b00667.
